# Morphodynamics of human early brain organoid development

**DOI:** 10.1038/s41586-025-09151-3

**Published:** 2025-06-18

**Authors:** Akanksha Jain, Gilles Gut, Fátima Sanchis-Calleja, Reto Tschannen, Zhisong He, Nicolas Luginbühl, Fides Zenk, Antonius Chrisnandy, Simon Streib, Christoph Harmel, Ryoko Okamoto, Malgorzata Santel, Makiko Seimiya, René Holtackers, Juliane K. Rohland, Sophie Martina Johanna Jansen, Matthias P. Lutolf, J. Gray Camp, Barbara Treutlein

**Affiliations:** 1 Department of Biosystems Science and Engineering, ETH Zürich, Basel, Switzerland; 2 Institute of Bioengineering, School of Life Sciences and School of Engineering, École Polytechnique Fédérale de Lausanne (EPFL), Lausanne, Switzerland; 3 Institute of Human Biology, Roche Pharma Research and Early Development, Roche Innovation Center, Basel, Switzerland; 4 Biozentrum, University of Basel, Basel, Switzerland

**Keywords:** Morphogenesis, Time-lapse imaging, Image processing, Multicellular systems, Neural stem cells

## Abstract

Brain organoids enable the mechanistic study of human brain development and provide opportunities to explore self-organization in unconstrained developmental systems^1–3^. Here we establish long-term, live light-sheet microscopy on unguided brain organoids generated from fluorescently labelled human induced pluripotent stem cells, which enables tracking of tissue morphology, cell behaviours and subcellular features over weeks of organoid development^4^. We provide a novel dual-channel, multi-mosaic and multi-protein labelling strategy combined with a computational demultiplexing approach to enable simultaneous quantification of distinct subcellular features during organoid development. We track actin, tubulin, plasma membrane, nucleus and nuclear envelope dynamics, and quantify cell morphometric and alignment changes during tissue-state transitions including neuroepithelial induction, maturation, lumenization and brain regionalization. On the basis of imaging and single-cell transcriptome modalities, we find that lumenal expansion and cell morphotype composition within the developing neuroepithelium are associated with modulation of gene expression programs involving extracellular matrix pathway regulators and mechanosensing. We show that an extrinsically provided matrix enhances lumen expansion as well as telencephalon formation, and unguided organoids grown in the absence of an extrinsic matrix have altered morphologies with increased neural crest and caudalized tissue identity. Matrix-induced regional guidance and lumen morphogenesis are linked to the WNT and Hippo (YAP1) signalling pathways, including spatially restricted induction of the WNT ligand secretion mediator (WLS) that marks the earliest emergence of non-telencephalic brain regions. Together, our work provides an inroad into studying human brain morphodynamics and supports a view that matrix-linked mechanosensing dynamics have a central role during brain regionalization.

## Abstract (web summary)

Human brain morphodynamics are explored using organoids.

## Main

Unguided human neural or brain organoids generated from pluripotent stem cells develop self-organized regionalized domains composed of cell types and states with remarkable structural and molecular similarities to primary tissue counterparts^2,5,6^. Unguided brain organoid development proceeds through assembly, self-patterning and morphogenetic mechanisms that reflect a latent intrinsic order emerging from the initial conditions of the system^7^. Multipotent embryoid bodies are directed towards the neuroectoderm, and the developing tissue can be supplied with an extrinsic matrix, such as Matrigel, that supports formation and expansion of a polarized neuroepithelium surrounding large luminal regions^8–10^. Regional domains form with different neural progenitor cell states that develop, proliferate and ultimately differentiate into diverse neuronal cell types^11^. Extracellular matrix (ECM) proteins and glycoproteins such as laminin, decorin and HAPLN1 are involved in many aspects of brain development^12^, and can be secreted from various cell types within and surrounding the developing brain (for example, neural progenitor cells and meningeal cells), thereby modifying extracellular microenvironments^13,14^. Much of what is known about ECM secretion and its role in brain development derives from studies in non-human model systems, and it has remained unclear how the extracellular microenvironment affects the early stages of human brain development. Organoid protocols exist that guide the development of specific brain regions by providing patterning molecules (morphogens such as BMP, SHH, FGF and SHH, among others) to the culture media^15–17^, and some of these protocols do not use an extrinsic ECM for the initial neuroectoderm formation^18–21^. It has been difficult to understand early brain organoid morphodynamics and the role of the extracellular microenvironment in shaping organoid morphogenetic patterning due to the lack of methods to dynamically track organoid development over many days. Recent advances in CRISPR-based stable fluorescent reporter tagging in stem cells and light-sheet microscopy are providing opportunities for in toto imaging of fluorescently labelled in vitro self-organizing systems^22–25^.

Current human brain organoid protocols pose challenges for live imaging because the organoids are relatively large in size, optically dense, slow in their development and require sterile imaging conditions for weeks to months of development. Here we address these challenges by developing a protocol for the generation of multi-mosaic, sparsely labelled brain organoids that are amenable to long-term live imaging, tracking and segmentation. We used this protocol, together with long-term light-sheet microscopy and a suite of computational tools, to study tissue morphodynamics, cellular behaviours and interactions with ECM over 2 weeks of organoid development. We quantified cell morphologies as pluripotent stem cells transition into a pseudostratified neuroepithelium, and observed interkinetic nuclear migrations, elongation of radial glial cells and differentiation into neurons. We found that exposure to an extrinsic ECM (Matrigel) modulates tissue morphogenesis by inducing cell polarization and neuroepithelial formation, fostering lumen enlargement through fusions, and altering the global patterning and regionalization of the organoids. These changes in tissue patterning are associated with modulation of the WNT signalling pathway and, in particular, YAP-mediated upregulation of WLS expression. Together, we have established a multiscale morphodynamic view of human brain organoid formation.

## Long-term live imaging of sparse and multi-mosaic fluorescently labelled brain organoids

**Fig. 1:**
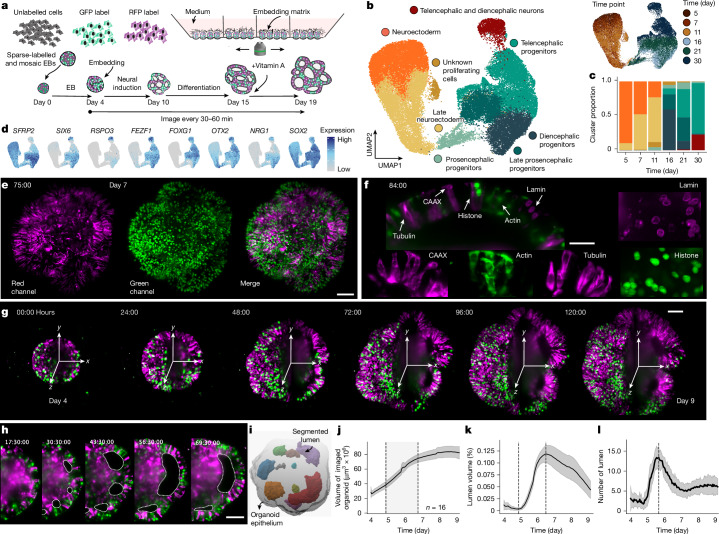
Long-term live imaging of sparse and multi-mosaic fluorescently labelled brain organoids. **a**, Schematic of the mosaic fluorescent organoid protocol and light-sheet image acquisition setup. EB, Embryoid body. **b**, UMAP embedding of organoid time-course scRNA-seq data with cells coloured by cluster and labelled by cell population (left) or time point (right). See Methods for cell numbers (*n*) at each timepoint. **c**, Stacked barplot showing the proportion of each cell population per time point. **d**, Feature plots showing normalized expression of representative marker genes. **e**, Maximum intensity projection image at 75 h of imaging from a 188-h imaging experiment. Organoids contain five different cell lines that contain stable genetic tagging of proteins with red or green fluorescent protein (RFP and GFP, respectively), as well as unlabelled cells. Scale bar, 100 µm. *n* = 16 organoids, imaged together. **f**, Organoid cross-section (84 h) showing nuclear membrane (lamin, RFP in magenta), plasma membrane label (CAAX, RFP in magenta), actin (GFP in green), tubulin (RFP in magenta) and nuclei (histone, GFP in green). Scale bar, 50 µm. **g**, Images of one organoid at different time points from a timecourse imaging experiment showing the maximum intensity projection (left) and cross-section (right). Scale bar, 100 µm. **h**, Cross-sections of an organoid showing lumen formation and fusion over time (hours). Dashed lines outline the lumen. Scale bar, 100 µm. 16 organoids, imaged together. **i**, 3D-rendered organoid showing segmented lumen and organoid epithelium masks. **j**, Graph showing total organoid volume measured per day from day 4 to day 9. **k**, Graph showing total volume of all lumen over time. **l**, Graph showing change in total number of segmented lumen over time. The dashed vertical line indicates the peak lumen number. The grey shading indicates standard deviation and the centre line denotes the mean (**j**–**l**). The dashed vertical lines in **j** and **k** denote the minimum (first line) and maximum (second line) of the lumen volume (%).

**Extended Data Fig. 1:**
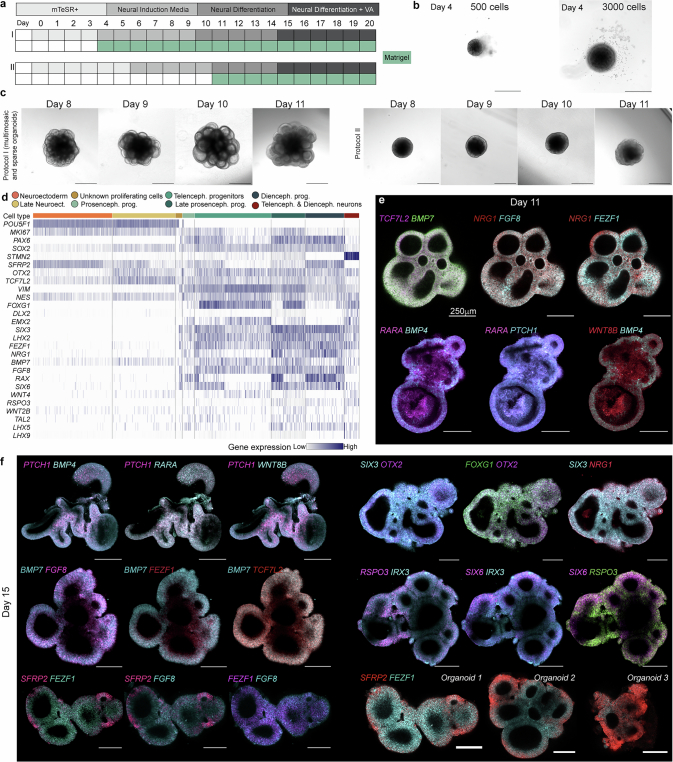
Protocol development for organoids compatible with long-term live light sheet imaging. **a**) Schematic of the protocol developed in this study (Protocol I) and a previous protocol to develop multi-region brain organoids (Protocol II)^2,26^. **b**) Comparison of organoid size at day 4 post aggregation at two cell seeding concentrations. Scale Bar is 500 micrometers. **c**) Brightfield images showing organoid growth from day 8–11 in the two different protocols. N = 3, n > 3. Scale Bar is 500 micrometers. **d**) Heatmap showing normalized marker gene expression grouped per cluster of organoid time-course scRNA-seq data shown in Fig. 1b. **e,f**) Images of example organoids stained with fluorescence in situ hybridization chain reaction (HCR) showing regionalized expression of marker genes in false color, merged using Fiji. Three organoids were used for staining in each case and the n indicates the number of organoids imaged. Organoids were stained whole-mount and images were acquired as z-stacks. Selected cross-sections after reorientation of the acquired volume are shown for each organoid. **e**) Day 11 (top n = 1), (bottom n = 3). **f**) Day 15 top left, n = 2, top right n = 3, middle left n = 1, middle right n = 3, bottom left n = 3. Scale, 250 micrometers in all images.

We established a protocol to generate sparse, mosaically labelled, fluorescent brain organoids that are amenable to long-term imaging using light-sheet fluorescence microscopy (Fig. 1a). Induced pluripotent stem cells (approximately 500 cells; see Methods; Supplementary Methods Table 1) containing genetic fluorescent labels were aggregated at day 0 into spherical embryoid bodies and cultured in medium maintaining proliferation and multipotency until day 4, when the organoids were transitioned into neural induction medium (NIM) containing extrinsic matrix (Matrigel). At day 10, media were exchanged to enhance neural differentiation, and at day 15, vitamin A was provided to support maturation. Compared with a previously published unguided brain organoid protocol^2,26^, the lower number of input cells and early exposure to matrix and neural induction led to organoids with a smaller initial size and earlier expansion of lumens surrounded by neuroepithelium (Extended Data Fig. 1a–c). Time-course single-cell transcriptomics (days 5, 7, 11, 16 and 21) revealed transitions from neuroectodermal progenitors (days 5–11) via early prosencephalic neural progenitors to regionalized neural progenitors (days 11–21) of predominantly telencephalon and diencephalon identity (Fig. 1b–d and Extended Data Fig. 1d). Whole-mount fluorescent in situ hybridization chain reaction (HCR) revealed spatial segregation of these developing brain regions (Extended Data Fig. 1e,f).

**Extended Data Fig. 2:**
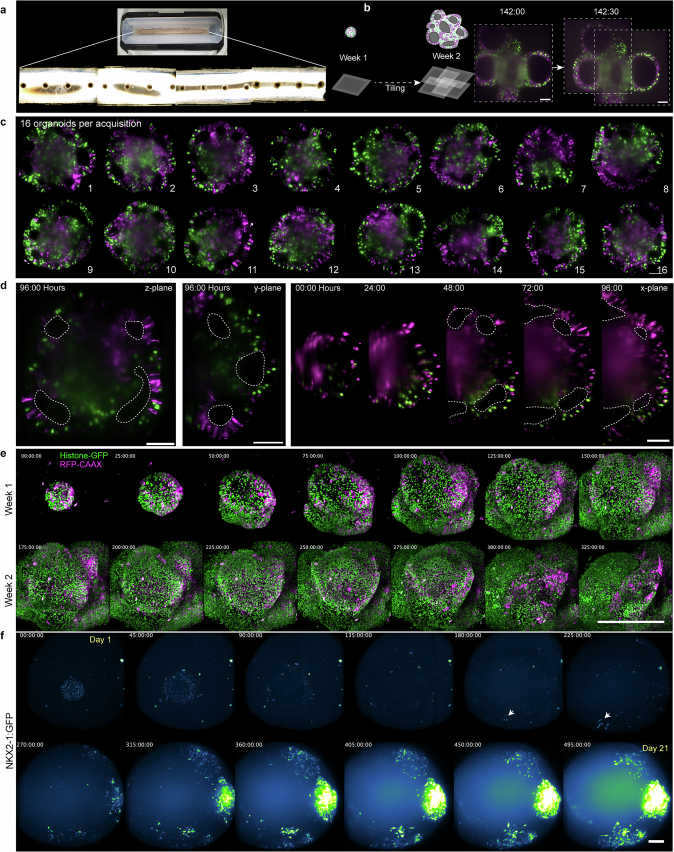
Long-term live imaging of fluorescent brain organoids. **a**) Photographs of the sample holding chamber with four separated sub-chambers each containing four microwells, for growing and imaging organoids with sixteen different organoids arranged one per microwell n = 16 organoids. **b**) Schematic overview of tiled image acquisition and example images showing two timepoints, before and after tiled acquisition and image fusion. Scale Bar is 100 micrometers. Time is in hours. n = 16 organoids. **c**) Cross section images (day 7) from 16 simultaneously imaged organoids, generated with cells lines labeled with nuclear membrane (lamin, RFP, magenta), plasma membrane (CAAX, RFP, magenta), actin (GFP, green), tubulin (RFP, magenta), and nuclei (histone, GFP, green) and unlabeled WTC-11. **d**) Images showing cross section (z-plane) and orthogonal views (y-plane, x-plane) of one organoid from a timecourse lightsheet imaging experiment shown in c. Scale Bar is 100 micrometers. Time is in hours. **e**) Maximum intensity projections from a 2-week imaging acquisition of a mosaic organoid (histone, GFP, green; CAAX, RFP, magenta), unlabeled WTC-11). Scale Bar is 500 micrometers. Time is in hours. n = 16 organoids. **f**) Images from a 3-week continuous imaging experiment using a NKX2-1:GFP reporter line. The organoids were given SHH morphogen treatment to induce ventral telencephalic patterning of the organoids. Images are false-colored with the green-fire-blue LUT. Scale Bar is 100 micrometers. Time is in hours. n = 4 organoids.

For live-imaging experiments, day 4 organoids were moved to the imaging chamber, covered with matrix to stabilize tissue location and provided with NIM (Fig. 1a and Extended Data Fig. 2a). This protocol enabled imaging for weeks of development using an inverted light-sheet platform with controlled environmental conditions suitable for in vitro cell culture applications (Viventis Microscopy sárl)^22,27^. The custom microscope was adapted for long-term brain organoid imaging with a 25× objective demagnified to 18.5× with a 710-μm field of view that captured the entire organoid during the first week of development, followed by tiling acquisition as the organoids grow larger (Extended Data Fig. 2b). We modified the sample mounting chamber to allow for stable long-term imaging over weeks, enabling medium exchanges with limited drift (Extended Data Fig. 2c–f). The custom sample chamber was composed of a fluorinated ethylene propylene bottom with rounded cone pockets of 800 µm diameter such that one organoid was added per microwell. The sample chamber was divided into four sub-chambers with vertical walls to separate different imaging conditions, in total containing four microwells per sub-chamber and enabling parallel imaging of up to 16 organoids in one experiment for 1–3 weeks (Extended Data Fig. 2a,c).

To explore cellular dynamics during organoid development, we used a set of induced pluripotent stem cell lines (based on WTC-11)^28^ each expressing a single endogenously tagged protein representing a particular organelle or cellular structure including the plasma membrane (CAAX, RFP), actin cytoskeleton (actin (ACTB), GFP), microtubules (tubulin (TUBA1B), RFP), nucleus (histone (HIST1H2BJ), GFP) and nuclear envelope (lamin (LAMB1), RFP). We combined these five labelled lines together with the unlabelled parental WTC-11 line at a ratio of 2:100 (labelled:unlabelled) to achieve sparse mosaicism for tracking and resolving single nuclei or cells for segmentation. This enables multiplexed profiling of the dynamics of multiple subcellular features in 3D, in each developing organoid (Fig. 1e,f and Extended Data Fig. 2d). Starting at day 4, organoids were imaged for 188 h with a 30-min time resolution to track 1 week of organoid development. This imaging timeframe followed organoids as they transitioned from spherical embryoid bodies to form a neuroepithelium composed of expanding lumens that began self-patterning at around 2 weeks (Fig. 1g and Supplementary Videos 1 and 2). Together, this approach provides a framework for imaging and exploring multiple phases of organoid development spanning neuroepithelial formation and brain regionalization.

**Extended Data Fig. 3:**
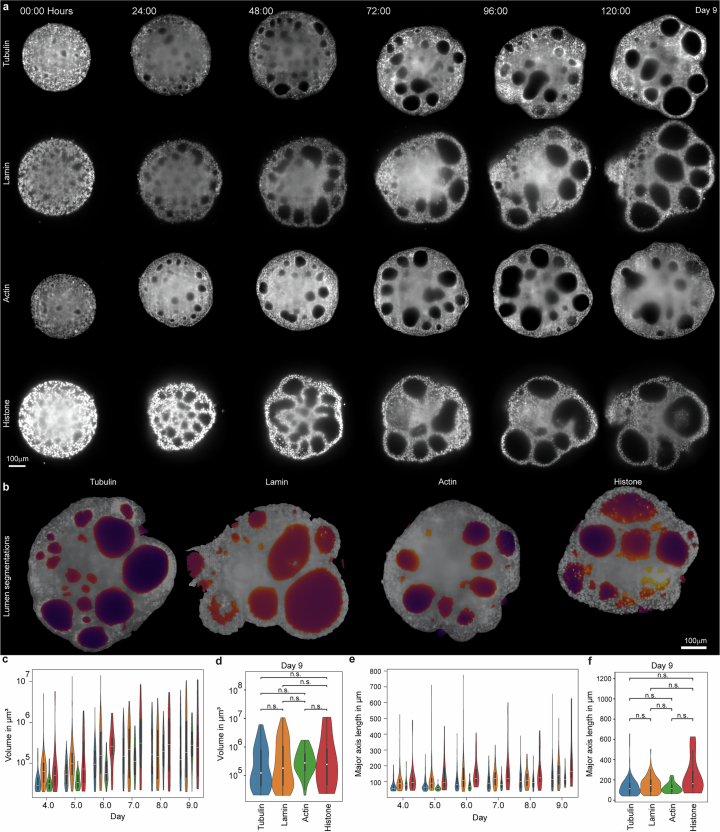
Lightsheet imaging and lumen segmentations to track tissue morphodynamics in organoid development. **a**) 2D cross sections from a time course lightsheet movie showing lumen development. Organoids were generated from 100% single label iPSC lines (histone2B-mEGFP mEGFP-beta-actin, mTagRFP–T–CAAX, mTagRFP–T-tubulin-alpha1b and mTagRFP–T-laminB1). Scale, 100 micrometers. Time is in hours. Three organoids were imaged for each label (n = 3). **b**) 2D cross sections from 3D imaged and segmented organoids shown in a, with organoid epithelium (gray) and segmented lumen (red) on day 9. Scale Bar is 100 micrometers. **c**) Violin plots showing 3D lumen volumes of organoids shown in **a**, over time. **d**) Violin plots showing lumen volumes on day 9, with horizontal bars indicating pairwise Wilcoxon rank sum test corrected for multiple testing using the Bonferroni method. P-values were > 0.5 for all comparisons. n.s. = not significant. **e**) Violin plots showing the major axis lengths of lumen over time. **f**) Violin plots showing the major axis lengths of lumen on day 9, with horizontal bars indicating Wilcoxon rank sum test corrected for multiple testing using the Bonferroni method. P-values were > 0.5 for all comparisons. n.s. = not significant. For p-values (d,f) see Supplementary Table 13. (c-f) The boxes of the violin plots show the interquartile range, the line at the center is the median and the whiskers extend to the data range excluding outliers.

**Extended Data Fig. 4:**
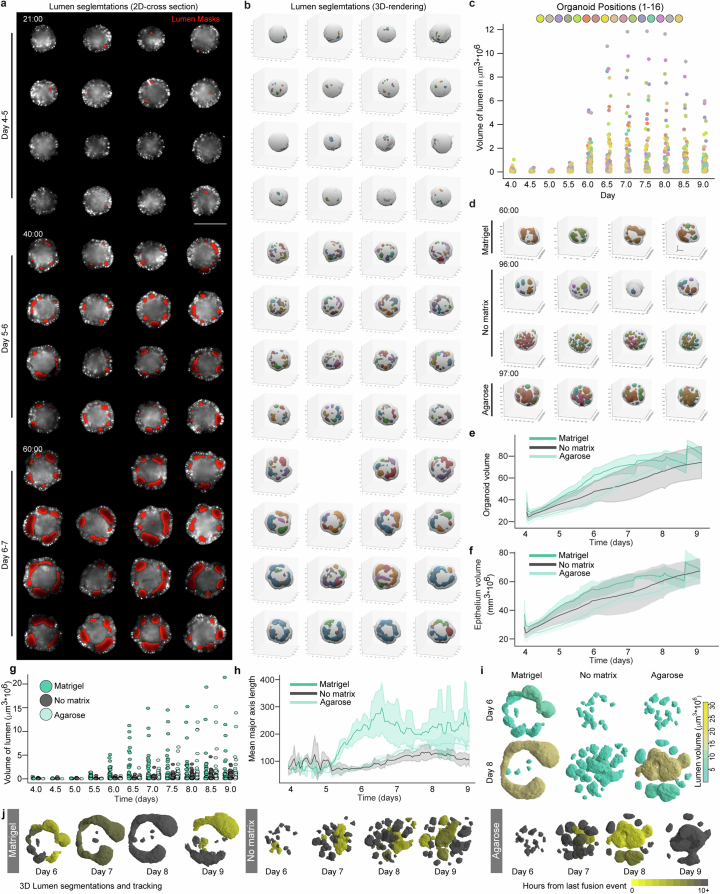
Lumen segmentations to track organoid and lumen morphodynamics in organoid development. **a**) 2D cross sections showing organoid epithelium (gray) and segmented lumen (red) from day 4–7. Scale, 500 micrometers. Time is in hours. All 16 organoids were imaged in one experiment. **b**) 3D renderings of segmented lumen for the corresponding organoids shown in **a**. Time is in hours. **c**) Plot shows individual lumen volumes over time, from day 4 to day 9, of all segmented lumen from organoids shown in **a** and **b**. **d**) 3D renderings of lumen segmentations and tracking. Lumens from the same track show the same color. 16 organoids were imaged in one experiment with 4 different organoids in matrigel condition, 8 organoids from no-matrix condition and 4 organoids for diffusion barrier condition. Time is in hours. **e-h**) Graphs showing total organoid volume (e), epithelium volume (f), individual lumen volumes (g) and mean major axis length measurements (h) of all segmented lumen for all imaged organoids in **d**. Error bars indicate standard deviation and the center line indicates the mean (e, f, h). Total imaged organoids used for measurements: Matrigel (n = 4), no-matrix (n = 8) and Diffusion barrier (n = 4). **i**) 3D renderings of segmented lumen in organoids shown in Fig. 2g, color coded for lumen volume. **j**) 3D renderings of segmented lumen in organoids shown in Fig. 2g, showing a timecourse of lumen development in Matrigel, no-matrix and agarose conditions. Lumen were segmented and tracked overtime. Color shows the hours from the last fusion event with yellow indicating the just fused lumen.

To quantify morphodynamic variation across organoids (multi-mosaic or single labelled), we imaged 16 organoids in parallel in a single imaging experiment (Extended Data Fig. 2c). After approximately 24 h of imaging, on day 5, we observed multiple cavitation spots in each organoid, which expanded over time into lumens surrounded by neuroepithelium (Fig. 1h and Extended Data Figs. 2d and 3a,b). We segmented and quantified tissue-scale properties such as organoid volume, lumen volume and lumen number per organoid to assess the tissue morphodynamics associated with neural induction, neuroepithelium formation and patterning of the organoids (Fig. 1i–l, Extended Data Figs. 3b,c and 4a–c and Supplementary Videos 3 and 4). Despite qualitative differences in morphology, we found that both single-labelled and multi-mosaic-labelled organoids exhibited consistent growth dynamics (Extended Data Fig. 3b–f). Between day 4 and day 8, organoids experienced a fourfold increase in overall volume (Fig. 1j), accompanied by an increase in total lumen volume from day 5 to day 8 (Fig. 1k). The average lumen number per organoid first increased from 3.7 ± 2.5 to 13.4 ± 2.5 between day 5 and day 6 and then decreased again to an average number of 5.4 lumens per organoid, indicating fusion of the small lumen (Fig. 1l). After day 7, the lumen number per organoid remained stable, whereas the lumen volume decreased (Fig. 1k,l and Supplementary Video 5). These observations highlight three morphodynamic phases of early brain organoid development including an early phase of rapid tissue and lumen growth, a phase of tissue stabilization involving lumen fusion events and finally a phase of neuroepithelium maturation.

## Extrinsic ECM affects brain organoid morphogenesis

**Fig. 2:**
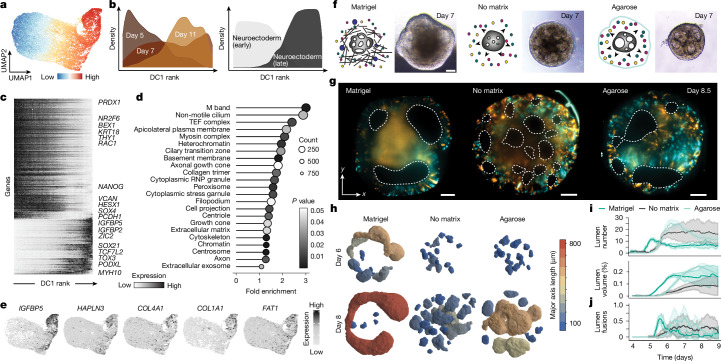
Extrinsic ECM affects brain organoid morphogenesis. **a**, UMAP embedding of scRNA-seq data with cells coloured by diffusion component ranking. **b**, Density plots showing cell distributions arranged in a pseudotime prediction (diffusion component 1 (DC1)) for time point (left) and cell type (right) labels. **c**, Heatmap showing normalized gene expression over DC1 ranking. **d**, Top DAVID Gene Ontology analysis terms calculated for genes that change over pseudotime from day 5 to day 11. A Fisher’s exact test was used to assess the significance of enrichment. **e**, Feature plots showing normalized expression of example ECM-related genes that show an increase in expression over time. **f**, Schematic representation of the extracellular microenvironment and the corresponding brightfield image for organoids grown with Matrigel (extrinsic ECM), without any external embedding (no-matrix) and with a low-melting agarose embedding (diffusion barrier). *N* = 3, *n* = 4 organoids. Scale bars, 100 µm. **g**, Images show cross-sections of sparse and multi-mosaic organoids containing cells labelled with nuclear membrane (lamin, RFP in orange), actin (GFP in cyan), tubulin (RFP in orange) and unlabelled cells from a light-sheet imaging experiment where organoids were embedded in Matrigel (*n* = 4), no-matrix (*n* = 8) or 0.6% agarose diffusion barrier (*n* = 4). The dashed lines outline the lumen. Scale bars, 100 µm. **h**, 3D renderings of segmented lumen in the organoids shown in panel **g**, colour coded for lumen axis measurements. **i**, Graphs showing total lumen number (top) measured per day from day 4 to day 9 for all imaged organoids, and total volume of all lumen (bottom) over time. **j**, Graph showing the number of lumen fusions over time. The shading indicates standard deviation and the centre line denotes the mean (**i**,**j**).

To understand the cell-state changes associated with these tissue transitions, we subsetted the single-cell RNA sequencing (scRNA-seq) data from days 5, 7 and 11 organoids, established a diffusion component-based pseudotemporal ordering of cells and explored pseudotime-dependent expression changes (Fig. 2a,b). We identified a major transcriptomic switch resolving the cell-state transition from an early neuroectoderm-like progenitor (*POU5F1*, *ITGA5*, *PROM1* and *THY1*) to a later neural tube-like neuroepithelial progenitor state (*SOX2*, *TCF7L2*,* OTX2*, *ZIC2*, *CYP26A1*,* ITGA6* and *SOX21*; Fig. 2c and Supplementary Tables 1 and 2). Genes that were upregulated over pseudotime showed a Gene Ontology enrichment for non-motile cilium, myosin complex and ECM-related terms (for example, basement membrane, collagen trimer and ECM; Fig. 2d,e and Supplementary Table 2). Genes upregulated over pseudotime included those encoding several ECM proteins (*COL1A1*, *COL11A1* and *LAMA5*) and ECM interactors (*ITGA6*, *HAPLN3*, *MMP16* and *IGFBP2*), some of which have been detected in primary neural progenitors^14,29,30^.

**Extended Data Fig. 5:**
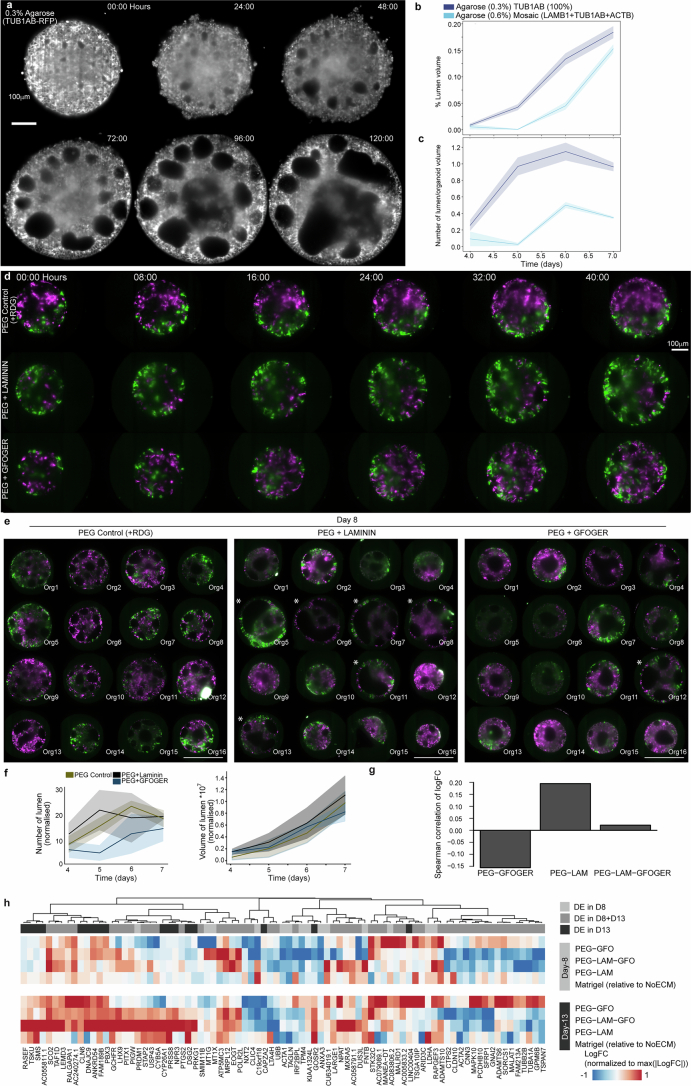
Mechanical and molecular differences in embedding matrix impact lumen morphogenesis and gene expression in developing organoids. **a**) 2D cross sections from a time course lightsheet movie showing lumen development. Organoids were generated from 100% single label iPSC line (mTagRFP–T-tubulin-alpha1b) and embedded in 0.3% Agarose. Scale, 100 micrometers. Time is in hours. **b,c**) Graphs showing % lumen volumes and number of lumen (normalised to organoid volume) of organoids shown in **a**, compared with multimosaic organoids (WTC-11, nuclear membrane (lamin, RFP, magenta), actin (GFP, green), tubulin (RFP, magenta)) embedded in 0.6% agarose shown in Fig. 2f. A single time point per day was segmented and used for quantifications. Error bars indicate standard deviation and the center line the mean, n = 4. **d**) 2D cross sections from a time course lightsheet movie showing lumen development in multi-mosaic labelled organoids (WTC-11, nuclear membrane (lamin, RFP, magenta), actin (GFP, green), tubulin (RFP, magenta)) that were embedded in PEG-RDG (control), PEG-LAM and PEG-GFOGER with controlled stiffness. n = 4 organoids for each condition. Scale, 100 micrometers. Time is in hours. **e**) 2D cross sections from organoids that were imaged on Day 8, using lightsheet showing lumen development in multi-mosaic labelled organoids (WTC-11, nuclear membrane (lamin, RFP, magenta), actin (GFP, green), tubulin (RFP, magenta)) that were embedded in PEG-RDG (control), PEG-LAM and PEG-GFOGER with controlled stiffness. 16 organoids from each condition were embedded in the same chamber and imaged. **f**) Graphs showing number of lumen and normalized lumen volumes of organoids shown in **d**. A single time point per day was segmented and used for quantifications. Error bars indicate standard deviation and the center line the mean, n = 4 for each condition. **g,h**) RNA sequencing analysis of organoids shown in **e** and used for a bulk RNAseq experiment. The bar plot shows the Spearman correlation coefficients between logFC of each PEG condition with PEG-RDG, and logFC of Matrigel relative to no-ECM, across highly variable genes in the samples (g). Heatmap showing genes upregulated in PEG-LAM, PEG-LAM-GFOGER and PEG-GFOGER compared with PEG-RDG and upregulated in Matrigel compared with no-matrix on day 8 and day 13 (h).

To assess the effect of ECM on tissue and cell-state transitions, we compared organoids cultured with Matrigel as a basement membrane-rich extrinsic matrix with organoids cultured without any extrinsic matrix or embedded in low-melting agarose (0.6% and 0.3%; Fig. 2f). Agarose is a polysaccharide gel that should provide an inert diffusion barrier to capture secreted ECM and other patterning molecules (morphogens) intrinsically produced from the organoid neuroepithelium^31^. Organoids grown without Matrigel were smaller and had different tissue morphologies lacking outgrowing neuroepithelium buds. We used long-term light-sheet microscopy to assess organoid morphodynamics and segmented and tracked lumen development over time across the three conditions (Fig. 2g, Extended Data Fig. 4d and Supplementary Videos 6 and 7). Organoids grown with Matrigel had larger organoid and epithelium volumes, formed lumen with a longer major axis with extended non-spherical shapes and showed peak lumen number on day 5.3 (Fig. 2h,i and Extended Data Fig. 4e–i). Organoids grown without an extrinsic matrix formed more lumen, had smaller organoid, epithelium and lumen volumes, rounded lumen and with peak lumen number on day 7.8 (Fig. 2h,i and Extended Data Fig. 4e–i). Peak lumen number (day 6.4) and lumen expansion were also delayed during growth within an inert diffusion barrier (agarose) compared with the Matrigel counterpart. We tracked lumen fusion events and found that organoids grown with Matrigel and agarose showed higher lumen fusion rates than organoids grown without an extrinsic matrix (Fig. 2j, Extended Data Fig. 4j and Supplementary Video 8). Reducing the agarose concentration from 0.6% to 0.3% changes the dynamics of lumen expansion (Extended Data Fig. 5a–c), suggesting that molecular composition as well as mechanical properties of the ECM affect lumen morphodynamics.

We used a synthetic hydrogel matrix based on polyethylene glycol (PEG) with a controlled stiffness (approximately 100 pa) that can be functionalized with ECM proteins to further assess the role of the ECM on lumen expansion. We compared multi-mosaic organoids cultured in a control PEG matrix (PEG-RDG), and organoids cultured in PEG containing purified laminin-111 (PEG-Lam), a collagen peptide (PEG-GFOGER) or both (Extended Data Fig. 5d–h). The organoids were imaged using light-sheet microscopy and transcriptomically characterized using RNA-seq. Organoids developed lumens in all conditions but showed different lumen morphologies and behaviours with PEG-GFOGER forming much fewer lumen (Extended Data Fig. 5d–f). Several organoids cultured in PEG-Lam formed expanding lumens with larger volumes similar to lumen behaviour in Matrigel (Extended Data Fig. 5e,f). Transcriptionally, PEG-Lam and PEG-LAM-GFOGER showed higher similarity to the Matrigel condition than PEG-GFO alone (Extended Data Fig. 5g). Several genes such as *TSKU*, *DSG2* and *CY26A1* were differentially upregulated in the presence of PEG-Lam and Matrigel compared with PEG-RDG and no-matrix, respectively (Extended Data Fig. 5h). Together, these data showed that the presence of extrinsic ECM has a major effect on tissue-scale morphogenesis in human brain organoids through alterations of lumen formation, expansion and fusion.

## Single-cell morphotype analysis reveals shape transitions within developing organoids

**Fig. 3:**
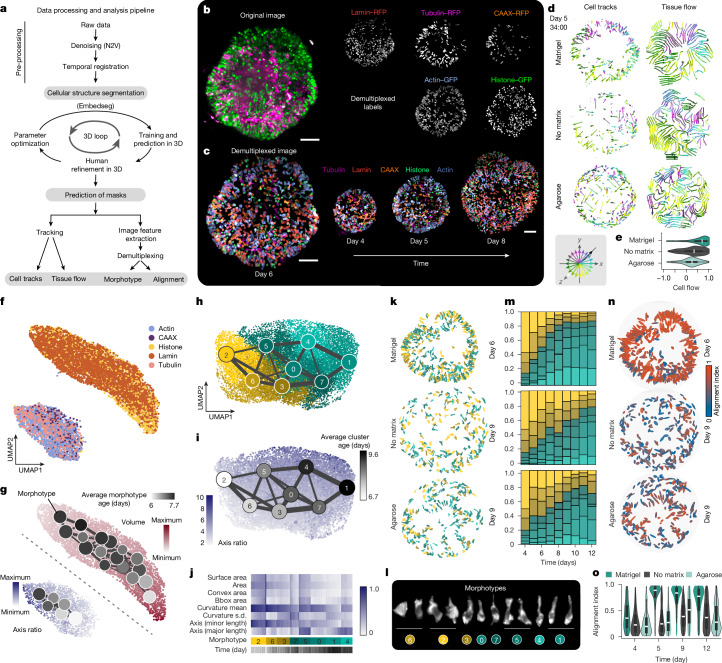
Cell and nucleus morphology transitions using demultiplexed mosaic cellular labels. **a**, Image analysis pipeline used for cell segmentation, demultiplexing and downstream analysis of mosaic cell labels. **b**, Maximum intensity projection image of an organoid at day 6 (left) showing dual-channel data (lamin, CAAX, tubulin (RFP, magenta), and actin, histone (GFP, green). Right: Corresponding demultiplexed images labelled with nuclear membrane (lamin, red), plasma membrane (CAAX, orange), actin (blue), tubulin (magenta) and nuclei (histone, green). Scale bar, 100 µm. **c**, Maximum intensity projection of demultiplexed image (lamin in red; CAAX in orange; actin in blue; tubulin in magenta; and histone in green). Scale bar, 100 µm. **d**, 4D cell tracks and tissue flows (averaged from tracks up to the last 24 h) during lumen expansion measured for all segmented actin-labelled cells from Matrigel, no-matrix and agarose conditions. The inset shows the colour key for arrow movements in 3D: white is towards the imaging objective and black is away from the imaging objective. **e**, Violin plot showing cell movements from the organoid centre towards the organoid surface (+1); *n* = 256 for Matrigel, 191 for no-matrix and 279 for agarose. **f**, PAGA initialized UMAP embeddings of all demultiplexed labels based on morphometric feature extraction. **g**, PAGA-initialized UMAP embeddings showing change in axis length for actin, tubulin and CAAX labels and change in nuclei volume measured using histone and lamin segmentations. PAGA plots show change in average cluster age (days), node size indicates the number of cells within one cluster, and edge width reflects the strength of connection between two clusters. **h**, PAGA-initialized UMAP embeddings and PAGA plots showing cell morphotype clusters using cells segmented from Matrigel, no-matrix and agarose conditions. The plots are based on morphometric measurements extracted for all segmented cells (actin). **i**, PAGA-initialized UMAP embeddings show a change in axis ratio of cells over time overlaid with average cluster age shown using PAGA plots. PAGA plots are colour coded based on the average age of the cluster from light grey to black. **j**, Heatmap showing example morphometric measurements for each morphotype cluster that are used to generate PAGA-initialized UMAP in panels **h**,**i**. **k**, Spatial distributions of actin-labelled cells in organoids showing cells coloured by their morphotype clusters. **l**, Example cells (actin) belonging to each of the morphotype clusters. **m**, Stacked barplots showing the proportion of cells in individual actin morphotype clusters in Matrigel, no-matrix and agarose conditions. **n**, All cells (actin) coloured by their alignment index (absolute cosine of the angle to the nearest organoid surface normal). Scale is from 0 to 1, with 1 (red) corresponding to cells that align perpendicular to the organoid surface. **o**, Violin plot showing the cell alignment (actin) values across all segmented cells from day 4 to day 12 for all three conditions. The boxes of the violin plots show the interquartile range, the line at the centre is the median and the whiskers extend to the data range excluding outliers (**e**,**o**).

**Extended Data Fig. 6:**
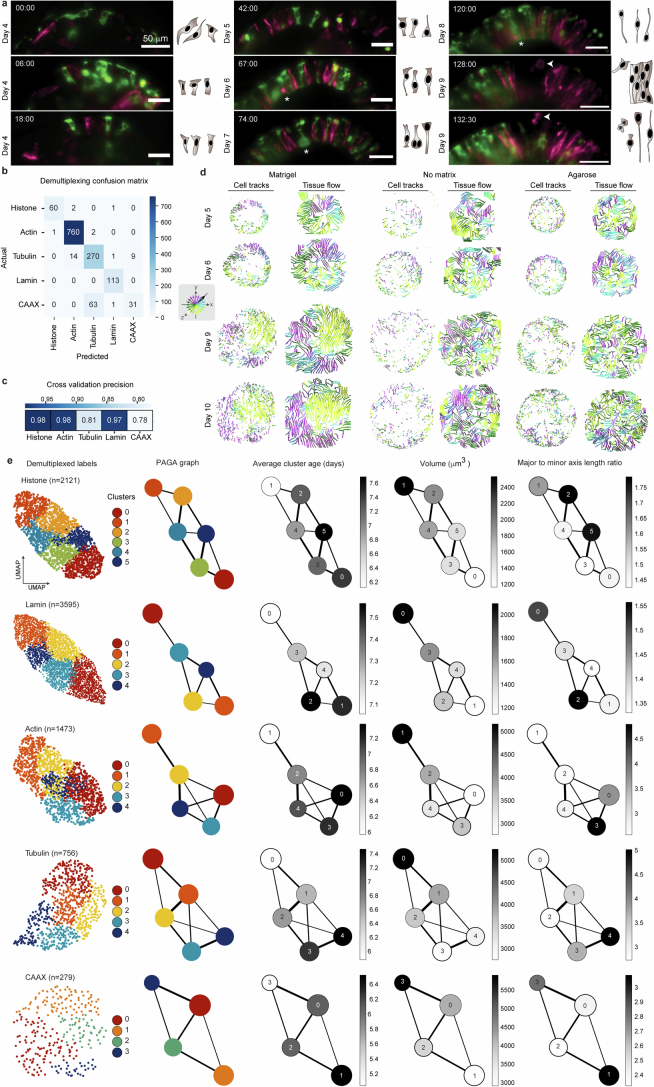
Demultiplexed labels reveal cellular morphodynamics. **a**) Cross-sections showing cell shape transitions from embryoid bodies to different stages of neuroepithelium formation and maturation in organoids from day 4 to day 9. Illustrations show different cell shapes seen in the cross-section images. Organoids are labeled with nuclear membrane (lamin, RFP, magenta), plasma membrane label (CAAX, RFP, magenta), actin (GFP, green), tubulin (RFP, magenta), and nuclei (histone, GFP, green). Asterisks mark cell division events seen in tubulin (magenta) and actin(green) labels. Arrowheads mark cell divisions on the basal surface of the organoids. Time is in hours. Scale, 50 micrometers. n = 16 organoids. **b**) Confusion matrix showing total number of demultiplexed cells assigned to each label and misclassifications between labels. **c**) The marker specific precision for demultiplexed labeling. **d**) 4D cell tracks and consequent tissue flows (averaged from tracks up to the last 24 h) for all segmented actin cells from Matrigel, no-matrix, and agarose conditions. **e**) UMAP embeddings and PAGA plots showing cell morphotype clusters of all 5 demultiplexed labels based on morphometric feature extraction. The PAGA plots per label show average cluster age, the change in nuclei volume and the change in axis length measured over time across all segmented cells. n number indicates the total number of cells per label from all timepoints.

We leveraged multi-mosaic labelling of plasma membrane, actin, tubulin, nuclear membrane and histone to assess cell morphology changes during early brain organoid development. We developed an image analysis pipeline that uses spatial embedding-based instance segmentation^32^ to predict cell and nucleus masks, and used morphometric feature extraction and a random forest classifier to demultiplex the fluorescence signals into the five individual labelled structures (Fig. 3a–c and Extended Data Fig. 6a–c). We tracked segmented cells and found that Matrigel-treated organoids exhibited coordinated cell and tissue flows towards the organoid surface corresponding to the initiation of lumen expansion and fusion events on day 5, whereas no-matrix and agarose organoids showed no coordinated cell or tissue flows, indicating that cells adopt very different trajectories in these conditions (Fig. 3d,e, Extended Data Fig. 6d and Supplementary Video 9). We generated a uniform manifold approximation and projection (UMAP) embedding based on extracted morphometric features with each point representing one cell structure. This revealed two major groups of nuclear (histone and lamin) and cellular (actin, tubulin and membrane) structures, capturing similarity in object morphologies (Fig. 3f). To quantify cell structural changes in the developing organoid, we performed high-resolution clustering to group structures into morphotypes (defined as a cluster of cells with similar morphological features such as volume, curvature and axis length), and used PAGA^33^ trajectory analysis to identify a gradient of structural changes in the tissue (Fig. 3g and Extended Data Fig. 6e). This revealed a time-dependent cytoskeletal and membrane elongation as well as nuclei compression (Fig. 3g and Extended Data Fig. 6e). Together, these analyses illuminate the morphological transition of multipotent stem cells to an early neuroectoderm and development into a matured neuroepithelium.

**Extended Data Fig. 7:**
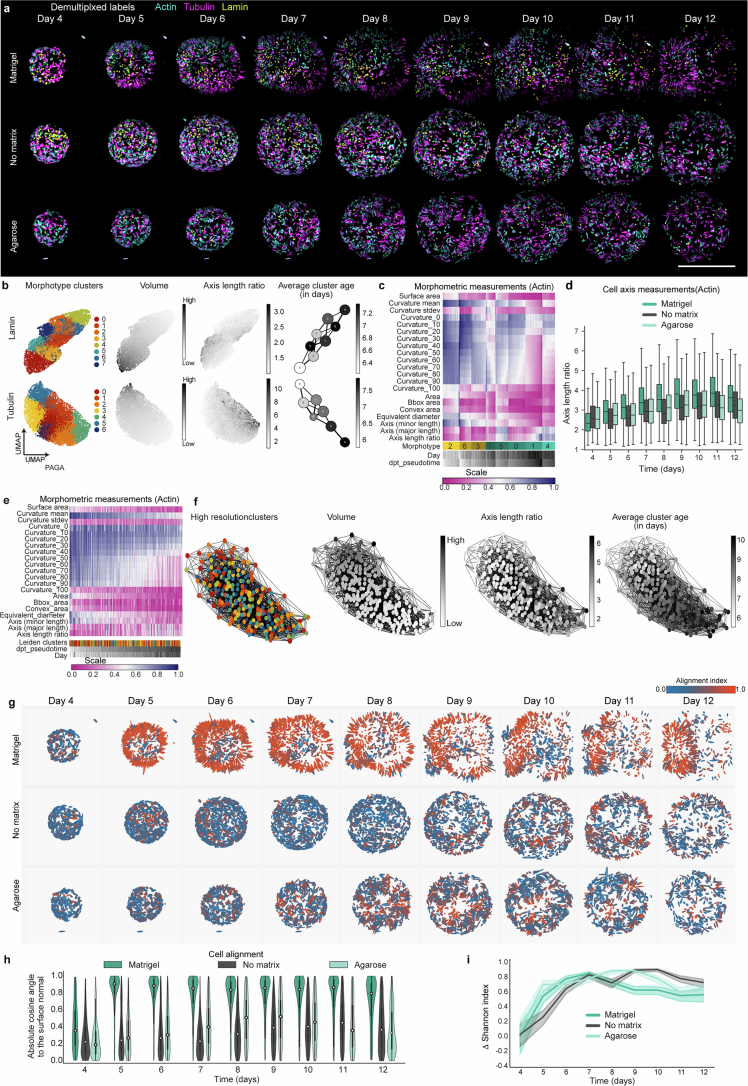
Demultiplexed labels reveal cellular morphodynamics in matrix perturbations. **a**) Demultiplexed images (lamin, yellow; actin, cyan; tubulin, magenta) of example organoids that were embedded with Matrigel, given no-matrix or embedded in agarose and imaged from day 4–12 in a light sheet microscope. Scale, 500 micrometers. **b**) UMAP embeddings showing clusters in lamin and tubulin labels based on morphometric feature extraction, change in axis length and volume measured using cell segmentations. PAGA plots show change in average cluster age (days). **c**) Extended heatmap from Fig. 3j, showing example morphometric measurements used for generating the PAGA derived UMAP shown in Fig. 3i. Day corresponds to real time and dpt_pseudotime is pseudotemporal ordering of cells in every morphotype cluster. **d**) Box plot showing axis length ratio per day in actin labeled cells across the 3 conditions, with the median shown, where the boxes show the quartiles of the measurement and the whiskers, the 1.5 IQRs of the lower and upper quartile. **e**) PAGA heatmap showing actin morphometric measurements derived from high resolution clustering. Day corresponds to real time and dpt_pseudotime is pseudotemporal ordering of cells in every morphotype cluster. **f**) PAGA plots showing high resolution clustering, volume, axis length ratio and average cluster age in days. **g**) Images segmented show cells (lamin, actin, tubulin) colored by their alignment index (absolute cosine of the angle to the nearest organoid surface normal) in all three conditions over time. Red colors indicate higher alignment (perpendicularity) to the cell surface. **h**) Violin plot showing the cell alignment values across all segmented cells (actin) from day 4 to day 12 for all three matrix conditions. The boxes of the violin plots show the interquartile range, the line at the center is the median and the whiskers extend to the data range excluding outliers. The n numbers (number of cells from all organoids per condition) are (Matrigel: day 4 to 12, n = 206, 462, 796, 865, 839, 871, 946, 778, 403), (No-matrix: day 4 to 12, n = 320, 521, 810, 732, 698, 791, 926, 872, 659), (agarose: day 4 to 12, n = 286, 454, 481, 547, 699, 684, 678, 557, 315)**. i**) Line plot showing the change in Shannon index between the three conditions based on change in the number of cells per cluster over time (organoids: Matrigel (n = 3), no-matrix (n = 3) and agarose (n = 3)) shown in Fig. 3m, with the error bars being the 95 percent confidence intervals and the center line the mean based on a bootstrapping analysis with 1000 repeats.

We next applied morphotype analysis to assess the effect of matrix perturbation on cell-shape transitions. We segmented labelled structures (actin, tubulin and lamin) at each imaging day from organoids in each matrix condition (Matrigel, agarose and no extrinsic matrix) and assessed morphotype heterogeneity focusing on a cytoskeletal label (actin; Fig. 3h–l and Extended Data Fig. 7a–f). We identified actin morphotypes that aligned along a temporal gradient corresponding to increasing cell elongation over time (Fig. 3h–j and Extended Data Fig. 7c–f). In the presence of Matrigel, organoids exhibited elongated morphotype clusters at an earlier time point than organoids grown in the absence of Matrigel (Fig. 3k–m). Cell axis and alignment (absolute cosine of the angle to the nearest organoid surface normal) quantification showed that cells align perpendicular to the organoid surface after exposure to Matrigel, whereas a small proportion of cells were aligned perpendicular to the surface in organoids without Matrigel (Fig. 3n,o and Extended Data Fig. 7g,h). Consistent with these observations, organoids grown without Matrigel showed a higher Shannon index (a diversity index), reflecting a more heterogeneous population of morphotypes (Extended Data Fig. 7i). We note that the presence of an inert diffusion barrier (agarose) induced higher cell alignment and cell elongation, and lower Shannon index than was seen in the absence of Matrigel (Fig. 3k–o). Together with the previous analysis of lumenal morphodynamics, these data show that organoids generated without Matrigel as an extrinsic ECM have alterations in tissue topology, contain a larger proportion of non-aligned and non-elongated cells, show higher heterogeneity in cell morphotypes and do not form a homogeneous neuroectoderm and neuroepithelium.

## Extrinsic matrix affects spatial patterning, cell identity and region emergence in organoids

**Fig. 4:**
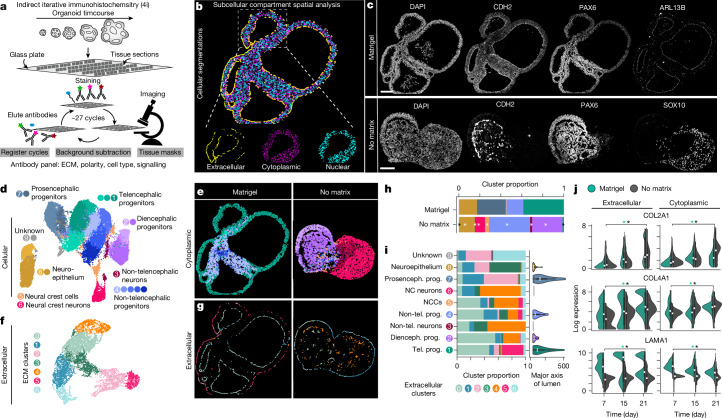
Multiplexed immunohistochemistry (4i) reveals spatial region emergence in organoid development. **a**, Overview of the 4i data acquisition pipeline. Organoids from a timecourse were fixed and sectioned followed by mounting on a glass coverslip. **b**, Image showing an example organoid section with segmented compartments (extracellular, cytoplasmic and nuclear) used for downstream quantitative analysis. **c**, Selected images showing protein stainings on day 21 on organoid slices in Matrigel (*n* = 4) and no-matrix (*n* = 3) conditions. **d**, UMAP embedding based on the combined cellular (nuclear + cytoplasmic) protein expression, with each dot representing a cell, clustered and annotated as distinct cell types. **e**, Example organoids (day 21) from each condition (Matrigel and no-matrix) with cell clusters projected back to the image. **f**, UMAP embedding based on the combined protein expression in the extracellular compartment showing individual clusters. **g**, Example organoids (day 21) from each condition (Matrigel and no-matrix) with extracellular cell clusters projected back to the image. **h**, Stacked barplot showing the cluster proportion of each cell population from all days in Matrigel and no-matrix conditions. *P < 0.05, calculated using a Fisher's exact test (two-sided) between the cluster proportions of the conditions corrected for multiple testing using the Benjamini–Hochberg method. For *P* values, see Supplementary Table 13. **i**, Stacked barplot showing the ECM cluster proportion per cell population. The violin plots show the major axis of the lumen that have been assigned to each cell cluster. Dienceph, diencephalic; NC, neural crest; NCC, neural crest cell; prog., progenitor; prosenceph., prosencephalic; tel., telencephalic. **j**, Violin plot showing the protein expression in extracellular and cellular compartments in Matrigel and no-matrix conditions. *P < 0.05, calculated using a one-way analysis of variance (ANOVA) across the timepoints for a condition corrected for multiple testing using the Benjamini–Hochberg method. *n* = 5,137 for Matrigel and *n* = 3,958 for no-matrix for the extracellular quantifications, and *n* = 17,140 for Matrigel and *n* = 16,007 for no-matrix for the cytoplasmic quantifications. The boxes of the violin plots show the interquartile range, the line at the centre is the median and the whiskers extend to the data range excluding outliers (**i**,**j**). For *P* values, see Supplementary Table 13. Scale bars, 100 µm (all panels).

**Extended Data Fig. 8:**
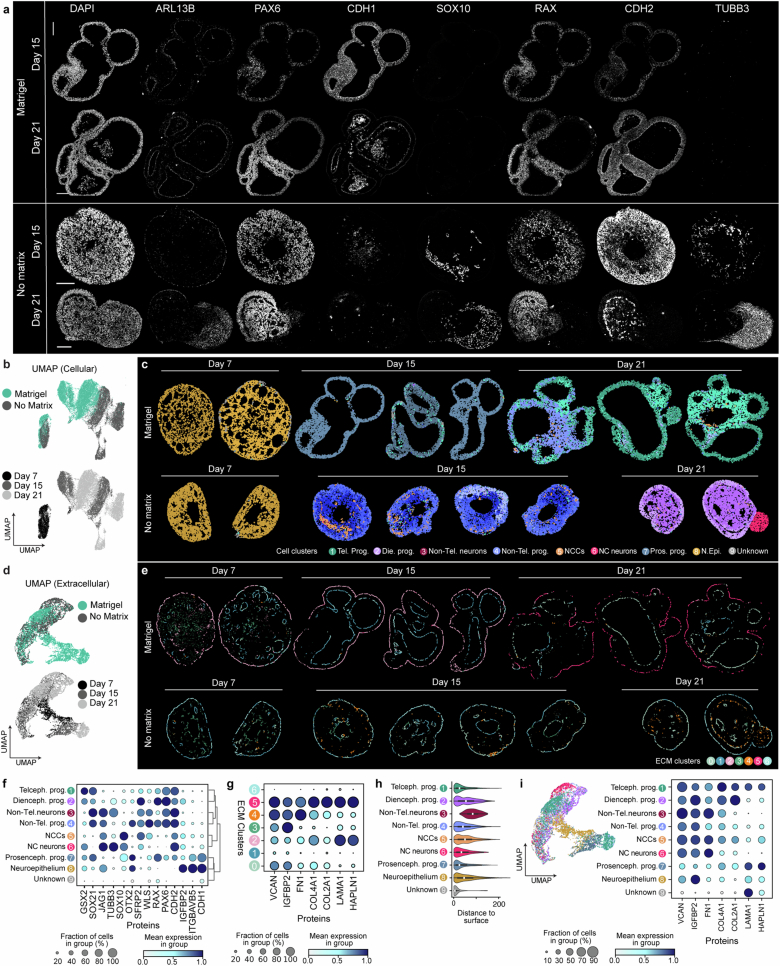
Multiplexed immunohistochemistry on matrix perturbations. **a**) Selected images show protein stainings on day 15 and 21 on organoid slices in Matrigel and no-matrix conditions, n numbers are added to c. **b**) UMAP embedding of multiplexed immunohistochemistry data (4i) based on cellular expression of proteins (Fig. 4d), with cells colored by treatment (Matrigel, no-matrix) and time (day 7, 15, 21). **c**) Spatial mapping of cell clusters in each organoid used for the combined analysis. The cell clusters are projected back to the original images from Matrigel and no-matrix conditions over the time course (day 7, 15, 21). Matrigel: day 7 n = 2, day 15 n = 3 and day 21 n = 4. No-matrix: day 7 n = 2, day 15 n = 4 and day 21 n = 3. **d**) UMAP embedding of multiplexed immunohistochemistry data (4i) based on the protein expression in the extracellular compartment of cells, with cells colored by treatment (Matrigel, no-matrix) and time (day 7, 15, 21). **e**) Spatial mapping of ECM clusters in each organoid used for the combined analysis. The ECM clusters are projected back to the original images from Matrigel and no-matrix conditions over the timecourse (day 7, 15, 21). **f**) Dotplot showing average expression and percentage of cells expressing selected regional marker proteins per cluster in b. **g**) Dotplot showing average expression and percentage of cells expressing ECM proteins per cluster in d. **h**) Violin plots showing the distance of each cell cluster from the organoid surface. The boxes in the violin plots show the interquartile range, with the line at the center as the median and the whiskers extending to the data range excluding outliers. **i**) UMAP embedding of multiplexed immunohistochemistry data (4i) based on the protein expression in the extracellular compartment of cells (shown in d, Fig. 4f), showing the distribution of cellular clusters shown in c and Fig. 4d. Right: Dotplot showing average expression and percentage of cells expressing ECM proteins in each cellular cluster. Scale, 100 micrometers in all images.

**Extended Data Fig. 9:**
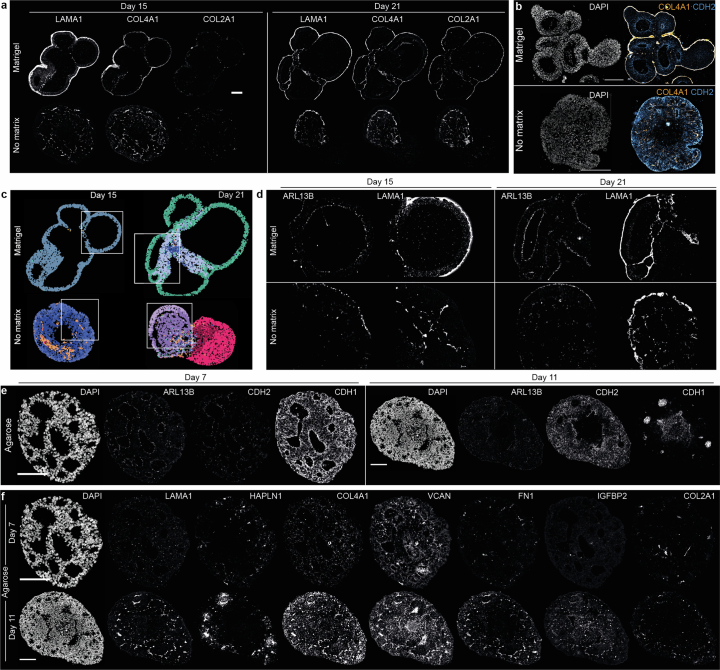
Multiplexed immunohistochemistry on matrix perturbations. **a**) Selected images show stainings of ECM proteins on day 15 and 21 on organoid slices in Matrigel and no-matrix conditions. **b**) Images show cross sections from an immunohistochemistry experiment on organoids (day 15) grown with Matrigel (n = 4) or with no-matrix (n = 4) and stained to show nuclei (DAPI), COL4A1 and CDH2. Scale Bar is 100 micrometers in all images. **c**) Example organoids from each condition (Matrigel, no-matrix) on day 15 and day 21 colored by cell clusters projected back to the image. Boxes highlight the luminal region from insets are shown in d. **d**) Images show lumen in organoids shown in c with ARL13B and LAMA1 protein expression marking neuroepithelium polarity. **e,f**) Images show protein stainings on organoid slices that were embedded in 0.6% agarose, fixed and stained in a 4i experiment. Day 7 (n = 2) and day 11 (n = 3). Images show polarity marker proteins (e) and ECM proteins (f). Scale, 100 micrometers in all images.

To analyse the effects of extrinsic matrix on organoid morphogenesis and patterning, we generated multiplexed spatial protein maps of early neural organoids grown in Matrigel, in agarose or without extrinsic matrix using iterative indirect immunohistochemistry imaging (4i)^34^ (Fig. 4 and Extended Data Figs. 8 and 9). We designed an antibody panel based on polarity markers (for example, CDH1, CDH2 and ARL13B), ECM proteins (for example, COL4A1, LAMA1, COL2A1 and FN1), cell-type and brain region markers (for example, RSPO3, SOX10, RAX and GSX2) and signalling pathway proteins (for example, WLS, SRFP2 and WNT5A; the full list is available in Supplementary Methods Table 2; Fig. 4a). Multiple organoids from Matrigel, agarose and no-matrix conditions over an early developmental time course (days 7, 15 and 21) were fixed, sectioned and stained for 27 cycles with three antibodies plus DAPI per cycle (Fig. 4c and Extended Data Fig. 8a). We developed a computational 4i analysis pipeline to segment images into extracellular, cytoplasmic and nuclear space and quantify protein abundances within these compartments (Fig. 4b). We clustered the cellular (nucleus and cytoplasmic) and extracellular compartment data of the Matrigel and no-matrix conditions and identified distinct cellular populations and extracellular clusters that we visualized in a UMAP embedding (Fig. 4d–g and Extended Data Fig. 8b,d,f,g). For the cellular compartment, we found neuroepithelial, neural progenitor and neuronal populations of different brain regions, as well as neural crest cells and neural crest-derived neurons. Mapping these populations to their respective tissue sections revealed their distinct location and temporal occurrence, indicating regional pattern emergence that is differential between the Matrigel and no-matrix conditions (Fig. 4e,h and Extended Data Fig. 8c,f). Day 7 organoids from both conditions were composed of neuroepithelial populations, whereas by day 15, Matrigel-exposed organoids showed large lumens surrounded by prosencephalic progenitors, and no-matrix organoids contained very few small lumens surrounded by non-telencephalic progenitors and neural crest cells (Fig. 4d,e and Extended Data Fig. 8c,f). At day 21, the periphery of Matrigel-exposed organoids was composed of telencephalic progenitors with a small proportion of non-telencephalic progenitors located in the centre of the organoid, whereas organoids without extrinsic matrix were composed of non-telencephalic (diencephalic) cells and neural crest-derived neurons and did not contain any telencephalic cells (Fig. 4d,e and Extended Data Fig. 8c,f). The Matrigel-exposed organoids were enriched in prosencephalic and telencephalic progenitors, whereas the no-matrix organoids formed non-telencephalic progenitors and neurons (Fig. 4h).

The extracellular compartment clusters also showed distinct location and temporal occurrence within the organoid tissue that differed between Matrigel and no-matrix conditions, similar to the cellular compartment clusters (Fig. 4f,g and Extended Data Fig. 8d,e,g). Specific extracellular clusters were associated with specific cellular populations and brain regional identities (Fig. 4i and Extended Data Fig. 8h,i). For example, extracellular clusters 2 and 5 had high expression of HAPLN1, LAMA1, COL2A1 and COL4A1 and co-occurred with prosencephalic and telencephalic cell clusters in Matrigel organoids, whereas extracellular cluster 4 had high expression of FN1, IGFBP2 and VCAN and was associated with non-telencephalic cell types found in no-matrix organoids (Fig. 4h,i and Extended Data Fig. 8g,i). Cellular and extracellular clusters also showed a correlation to lumen morphology with lumen assigned to telencephalic and prosencephalic populations, associated with extracellular clusters 2 and 5, found closer to organoid surface and exhibiting larger major axes (Fig. 4i and Extended Data Fig. 8h). In the presence of Matrigel, ECM proteins increased in abundance in both the extracellular and the cellular compartments and showed enrichment around the periphery of the organoid (Fig. 4j and Extended Data Fig. 9a–d). Together with the restricted expression of apical markers such as CDH2, ARL13B and CDH1 at the luminal surface, this indicates that the organoid neuroepithelium exhibits apicobasal polarity and directional ECM deposition (Fig. 4c and Extended Data Fig. 9a–d). By contrast, neuroepithelial tissue in organoids grown without any extrinsic matrix showed either inverted polarity with deposition of ECM proteins on the inside of the organoid, or a mixed phenotype with both inner and peripheral accumulation of polarity markers (Fig. 4c and Extended Data Fig. 9a,b,d). Of note, organoids embedded in agarose contain luminal domains with apicobasal polarity and ECM deposition within luminal regions but lack a surrounding basement membrane (Extended Data Fig. 9e,f). Together, we found that an external basement membrane surrounding the organoid promotes lumen morphogenesis and rostral patterning, and that ECM proteins exhibit regional heterogeneity in their expression associated with different brain regions.

## Matrix affects organoid morphogenesis and patterning through WNT pathway modulation

**Extended Data Fig. 10:**
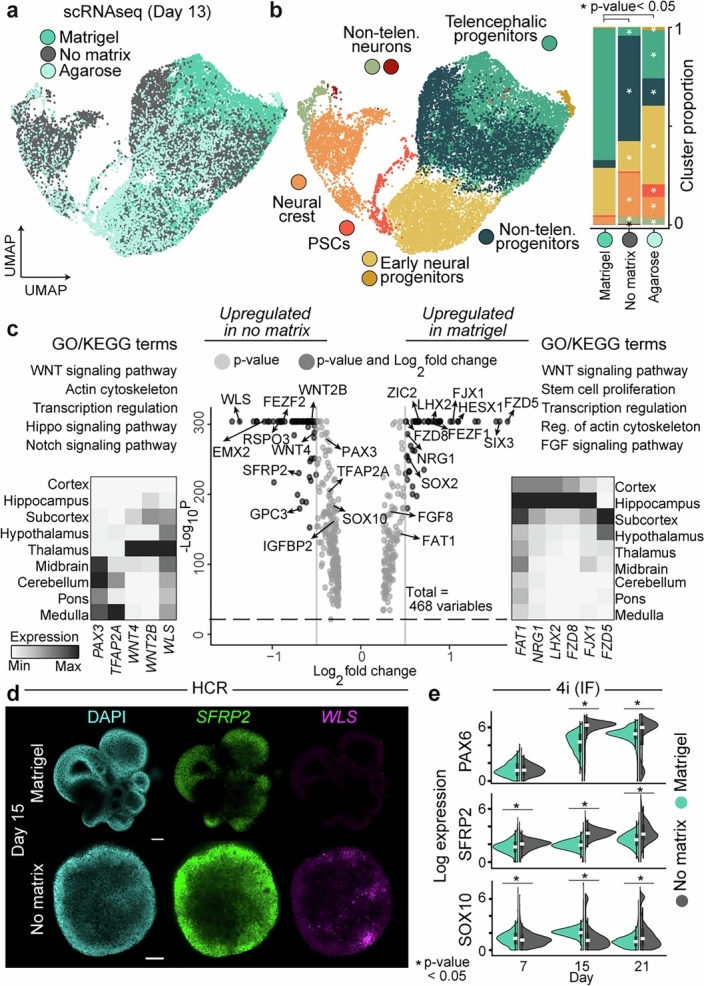
Matrix impacts organoid patterning and regionalization. **a-b**) UMAP embedding of scRNA-seq data with cells colored by condition (a) and cell population (b). Stacked barplot shows cluster proportion per condition. Abbreviations: Non-telen. = Non telencephalic, PSCs = Pluripotent stem cells. A star indicates p < 0.05, calculated using a Fisher’s exact test between the cluster proportions of Matrigel and no-matrix or agarose conditions, corrected for multiple testing using the Benjamini-Hochberg method. For p-values see Supplementary Table 13. **c**) Volcano plot highlights differentially expressed genes between Matrigel and no-matrix conditions calculated using a non-parametric Wilcoxon Rank Sum test with p-value cutoff using adjusted p-value. The p-value adjustment was performed using Bonferroni correction based on the total number of genes in the dataset. Positive and negative log fold change indicates genes with higher expression in Matrigel or higher expression in no-matrix conditions, respectively. GO/KEGG terms enriched for genes differentially upregulated between Matrigel (right) and no-matrix (left) conditions are shown. Heatmaps show average gene expression of selected differentially expressed genes in different regions of the human fetal brain tissue from 5-14 post-conceptional weeks^35^. **d**) Cross sections from a whole mount fluorescence in situ hybridization chain reaction (HCR) staining for selected differentially expressed genes between Matrigel and no-matrix conditions. Scale, 100 micrometers. n = 3. **e**) Violin plots showing the total protein expression in Matrigel and no-matrix organoids over time and calculated from the 4i experiment shown in Fig. 4 and Extended Data Figs. 8 and 9. The boxes of the violin plots show the interquartile range, the line at the center is the median and the whiskers extend to the data range excluding outliers. A star indicates p < 0.05, calculated using a Wilcoxon rank sum test (two-sided) between conditions corrected for multiple testing using the Benjamini-Hochberg method. For p-values see Supplementary Table 13. n = 5289 (Day 7), n = 11690 (Day 15), n = 16168 (Day 21).

**Extended Data Fig. 11:**
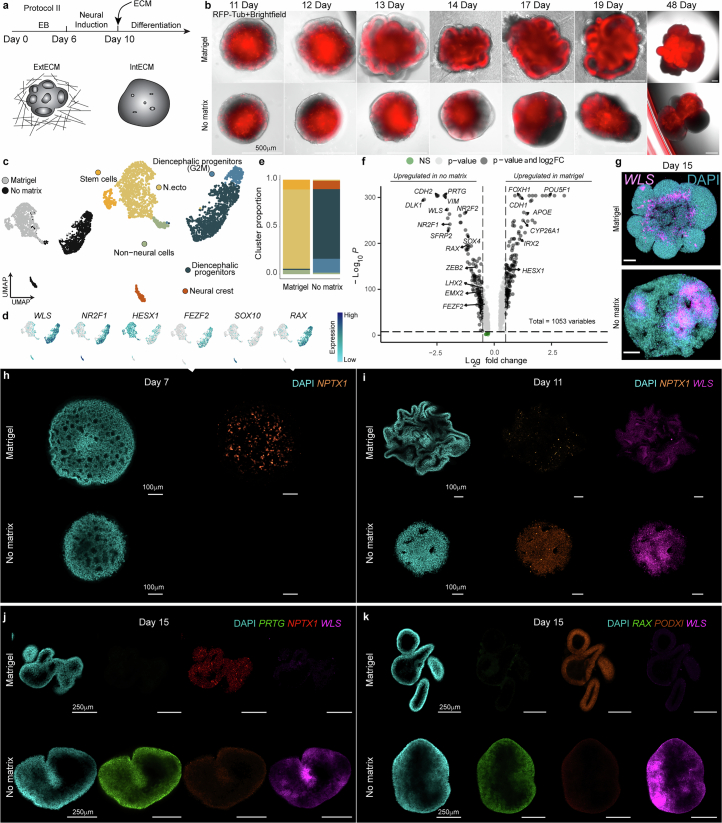
Matrigel influences organoid regional patterning. **a**) Overview of the protocol used to generate organoids to test the influence of matrix on brain organoids development (Cerebral organoid, Protocol II). **b**) Time-course images of organoids generated from mTagRFP–T-Tubulin-alpha1b iPSC line, showing merged brightfield and RFP expression in the presence (n = 4) and absence (n = 2) of Matrigel. Imaging was done with a widefield Nikon Ti2 microscope. **c**) UMAP embeddings of scRNA-seq data performed at day 16 with cells labeled by treatment (left) or colored by cluster and labeled by cell population (right). **d**) Feature plots showing normalized expression of selected marker genes. **e**) barplot showing cluster proportions of the cell populations in Matrigel and no-matrix organoids. **f**) Volcano plot showing differentially expressed genes upregulated in Matrigel (right) and upregulated in no-matrix conditions (left), calculated using a non-parametric Wilcoxon Rank Sum test with p-value cutoff using adjusted p-value. The p-value adjustment was performed using Bonferroni correction based on the total number of genes in the dataset. **g-k**) Images show example organoids stained whole-mount with fluorescence in situ hybridization chain reaction (HCR) showing regionalized expression of marker genes (false color, merged using Fiji) between organoids grown with Matrigel or with no-matrix. Three organoids were used for staining in each case. The n indicates the number of organoids imaged. **g**) Images show maximum intensity projections of organoids treated with Matrigel (top, n = 2) and or with no-matrix (bottom, n = 1) on day 15. Organoids were generated with protocol II, shown in **a**. Scale, 100 micrometers. **h-k**) Cross section images of HCR stainings. **h**) Day 7, Matrigel n = 1 and no-matrix n = 2. **i**) Day 11, n = 3 for both. **j**) Day 15 n = 3 for both. **k**) Day 15 no-matrix n = 2 and Matrigel n = 3. Organoids were generated with protocol shown in Fig. 1a. Scale, 250 micrometers.

To further understand the molecular processes affected by the different matrix conditions, we performed single-cell transcriptomic analysis of organoids grown in Matrigel, agarose or without extrinsic matrix at day 13 of organoid development (Extended Data Figs. 10a–c and 11a–f). Consistent with our 4i data, scRNA-seq analysis identified diverse cell populations including telencephalic and non-telencephalic neural progenitors, as well as neural crest cells and peripheral nervous system neurons. The proportion of each population differed between matrix conditions, with organoids grown in the presence of Matrigel containing significantly more telencephalic progenitors and fewer neural crest cells than no-matrix or agarose-exposed organoids (*P* < 0.05; Extended Data Fig. 10a,b). Gene Ontology analysis on differentially expressed genes between Matrigel and no-matrix conditions showed enrichments for multiple signalling pathways, including components of WNT, Notch, FGF and Hippo signalling, as well as genes associated with actin cytoskeleton regulation (Extended Data Fig. 10c and Supplementary Tables 3–6). Consistent with the enriched proportion of telencephalic progenitors, multiple transcription factors associated with rostral neural tube fate (for example, *SIX3*, *LHX2*, *NRG1*, *FOXH1* and *HESX1*) showed higher expression in Matrigel-exposed organoids. We identified *WLS*, *RSPO3* and *GPC3* as highly upregulated genes in organoids grown without extrinsic matrix, indicating that the WNT–β-catenin signalling pathway might be upregulated in this condition (Extended Data Figs. 10 and 11c–f). *WLS* has been previously identified as one of the earliest markers of non-telencephalic fate in human brain organoids^6^, and *WLS* as well as other WNT-associated genes upregulated in the no-matrix condition are highly expressed in non-telencephalic cells in the primary human developing brain^35^ (Extended Data Fig. 10c). We confirmed the differential expression of *WLS*, *SFRP2*, *NPTX1*, *PRTG*, *PODXL* and *RAX*, as well as PAX6, SFRP2 and SOX10 between Matrigel and no-matrix conditions using whole-mount HCR staining and immunohistochemistry (4i)-based quantifications, respectively (Extended Data Figs. 10d,e and 11g–k). Together, these data indicate that the matrix regulates dorsoventral and rostrocaudal patterning of brain organoids through modulation of the WNT signalling pathway.

## YAP1-mediated WLS activation affects brain organoid morphogenesis and regional patterning

**Fig. 5:**
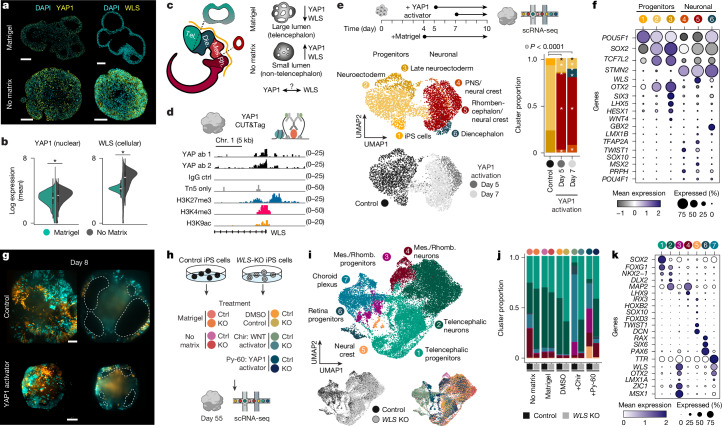
YAP1 mechanotransduction-mediated WLS activation. **a**, Images show cross-sections of organoids stained with antibodies labelling YAP1 (day 16) and WLS (day 15) from Matrigel and no-matrix conditions. Scale bars, 100 µm. **b**, Violin plots showing protein expression distribution of nuclear YAP1 (day 16) and cytoplasmic WLS (day 15) from Matrigel and no-matrix conditions. *P < 0.05, calculated using a Wilcoxon rank-sum test (two-sided) between conditions corrected for multiple testing using the Benjamini–Hochberg method. The boxes of the violin plots show the interquartile range, the line at the centre is the median and the whiskers extend to the data range excluding outliers. For *P* values, see Supplementary Table 13. **c**, Schematic showing the developing brain with distinct regions along the rostrocaudal axis: prosencephalon (telencephalon (Tel.) + diencephalon (Die.)), mesencephalon (Mes.) and rhombencephalon (Rh.). The dotted lines show coronal sections to illustrate lumen (brain ventricle) size differences. A schematic summarizing the morphological distinctions between Matrigel and no-matrix organoids with corresponding YAP1 and WLS expression differences is also shown (right). **d**, Signal tracks of bulk CUT&Tag sequencing data showing the enrichment intensity of YAP1 binding to the *WLS* gene, profiled with two different YAP1 antibodies. Tracks are shown for IgG and Tn5 control and the repressive and active marks profiled with H3K27me3, H3K4me3 and H3K9ac antibodies. Chr. 1, chromosome 1. **e**, Schematic of the light-sheet imaging and scRNA-seq experiment with control and YAP1 activator-treated organoids (top). EBs were cultured in NIM with Matrigel embedding on day 4. YAP1 activator (Py-60) or DMSO (control) was added to the imaging sub-chamber on day 5 or day 7. Imaging was terminated on day 10, and corresponding organoids from all three conditions were profiled with scRNA-seq on day 10. UMAP embeddings of scRNA-seq data from day 10 organoids in control and YAP1 treatment conditions are also shown (bottom left). A stacked barplot showing the cluster proportion of each cell population is also shown (bottom right). *P < 0.05, calculated using a Fisher’s exact test between the cluster proportions of the control and day 5-treated or day 7-treated conditions corrected for multiple testing using the Benjamini–Hochberg method. For *P* values, see Supplementary Table 13. The number of cells recovered after pre-processing of the scRNA-seq experiment: *n* = 2,085 for control, *n* = 763 for Py-60 on day 5 and *n* = 1,955 for Py-60 on day 7. **f**, Dotplot showing average expression and percentage of cells expressing selected regional marker genes per cell population. **g**, Maximum intensity projections (left) and cross-sections (right) at day 8, showing control organoids and YAP1 activator (given on day 5) treated organoids imaged with light-sheet microscopy. Sparse and multi-mosaic organoids contain cells labelled with nuclear membrane (lamin, RFP in orange), actin (GFP in cyan) and tubulin (RFP in orange) and unlabelled cells. Scale bars, 100 µm. Organoids imaged per condition, *n* = 4. **h**, Schematic of the scRNA-seq experiment with organoids generated from control and *WLS*-knockout (*WLS*-KO) iPS cell lines with five treatments. EBs were cultured in Matrigel or no-matrix conditions starting at day 4. WNT (CHIR99021) or YAP1 (Py-60) activators were added to a subset of organoids cultured with Matrigel from day 10 to day 12 and day 10 to day 16, respectively. Organoids were hashed and profiled with scRNA-seq on day 55. **i**, UMAP embeddings of scRNA-seq data coloured by cell population (top), genetic status (bottom left) or condition (bottom right). Mes./rhomb., mesencephalon or rhombencephalon. **j**, Stacked barplot showing the cluster proportion of each cell population in the different treatment conditions. **k**, Dotplot showing the average expression and the percentage of cells expressing selected regional marker genes per cell populations.

**Extended Data Fig. 12:**
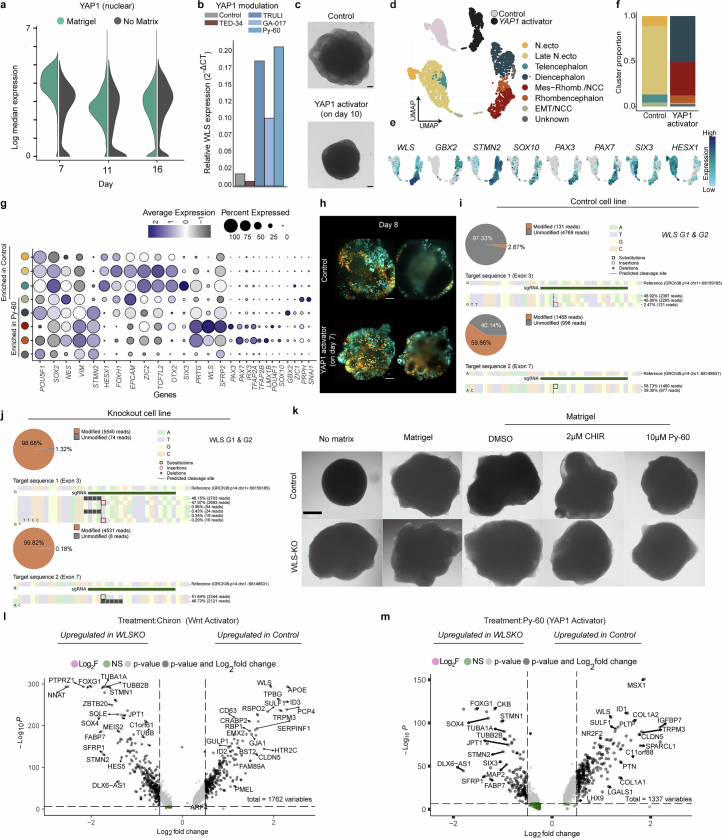
YAP1 and WLS influence organoid patterning. **a**) Violin plots showing the expression of nuclear YAP1 in organoids over time (immunohistochemical staining quantification). (Matrigel number of nuclei (days 7,11,16): n = 4631, 5104, 13824, no-matrix (days 7,11,16): n = 3390, 5023, 11025). **b**) Barplot shows relative *WLS* expression in no-matrix organoids on day 15 measured using qPCR, averaged from 3 replicates, N = 1. Organoids were treated with YAP1 activators (TRULI, GA-017, Py-60) and inhibitor (TED-34) on day 10. **c**) Brightfield images at day 13, showing a control organoid (n = 5) and an organoid cultured with YAP1 activator (10 µM Py-60) together with Matrigel (n = 13) from day 10 onwards. Organoid protocol is shown in Extended Data Fig. 11a. Scale, 100 micrometers. **d**) UMAP embedding of scRNA-seq data from 16-day old organoids with cells colored by treatment (top) and cluster and labeled by cell population (bottom). Abbreviations: N.ecto = neuroectoderm, NCC = neural crest cells and EMT = epithelial-to-mesenchymal transition. **e**) Feature Plots showing normalized expression of marker genes. **f**) Stacked barplot showing the proportion of each cell population in control and YAP1 activator treatment. **g**) Dotplot showing average expression and percentage of cells expressing selected regional marker genes used for annotating the cell populations shown in **d**. **h**) Maximum intensity projections and cross-section images at day 8 showing control and YAP1 activator (given on day 7) treated organoids imaged with lightsheet microscopy. Sparse and multi-mosaic organoids contain cells labeled with nuclear membrane (lamin, RFP, orange), actin (GFP, cyan) and tubulin (RFP, orange) and unlabeled cells. Scale, 100 micrometers. n = 4. **i,j**) Snapshots of CRISPResso2 readout showing quantification of editing frequency as determined by the percentage and number of reads showing unmodified and modified alleles for the control (unedited) and WLS-KO cell lines. **k**) Brightfield images showing 54-day old organoids generated from control and *WLS*-KO cell lines and given different treatments. The organoids were used for single-cell RNAseq on day 55 (Fig. 5h–k). Scale, 500 micrometers. n = 12 for Matrigel and no-matrix, n = 8 for DMSO control, Py-60 and CHIR99021 treatments **l,m**) Volcano plot highlights differentially expressed genes between Matrigel and no-matrix conditions from organoids treated with Chiron (l) or Py-60 (m). Positive and negative log fold change indicates genes with higher expression in Matrigel or higher expression in no-matrix conditions, respectively. The differential gene expression was calculated using a non-parametric Wilcoxon Rank Sum test with p-value cutoff using adjusted p-value. The p-value adjustment was performed using Bonferroni correction based on the total number of genes in the dataset.

YAP1 showed increased expression along with higher levels of WLS in organoids grown without extrinsic matrix than Matrigel-exposed organoids after 2 weeks of development (Fig. 5a,b and Extended Data Fig. 12a). YAP1, a mechanotransducer involved in sensing the mechanical properties in tissues, has recently been reported to upregulate WNT pathway genes including *WLS* in cardiomyocytes^36,37^ and to mediate crosstalk between WNT and Hippo signalling pathways^38^. During brain development, WNT signalling is known to regulate rostrocaudal patterning^39–41^ (Fig. 5c). We therefore wondered whether *WLS* upregulation in brain organoids grown without matrix is mediated by YAP1 and whether *WLS* is essential for neural organoid caudalization (Fig. 5c). We mapped YAP1 binding to the genome using CUT&Tag in early developing brain organoids and observed direct binding of YAP1 to the *WLS* promoter region (Fig. 5d). We further activated (Py-60, TRULI and GA-017)^42–44^ and inhibited (TED-347)^45^ the YAP1–Hippo pathway in early developing organoids and observed robust upregulation and downregulation of *WLS* expression, respectively (Extended Data Fig. 12b). We next performed scRNA-seq after YAP1 pathway activation (Py-60) on day 10 organoids treated for 3 or 5 days before sequencing (Fig. 5e). YAP1 activation led to a significantly different organoid cell composition with a higher proportion of non-telencephalic neuronal cells (clusters 4–6), whereas control organoids were predominantly still in a neuroectodermal progenitor state (*P* < 0.05; Fig. 5e). Genes upregulated upon YAP1 activation included *WLS*, *TFAP2A*, *MSX2* and *PRTG*, whereas genes expressed higher in control cells included *ZIC2*, *OTX2*, *IGFBP2* and *FOXH1* (Fig. 5f and Supplementary Tables 7 and 8). In addition, we found expression of *GBX2*, *LMX1B*, *PRPH* and *STMN2* in the YAP1 activation condition, consistent with caudalization of brain organoid tissue and premature neuronal differentiation (Fig. 5f). We confirmed the upregulation of WLS and premature differentiation upon YAP1 activation in a separate scRNA-seq experiment in which we treated organoids with Py-60 from day 10 to day 16 (Extended Data Fig. 12c–g). Finally, we explored the link between YAP1 activation and lumen morphogenesis using light-sheet imaging. Organoids grown in Matrigel with YAP1 activator treatment exhibited an inability to expand and maintain lumens, resulting in altered organoid and lumen morphologies (Fig. 5g, Extended Data Fig. 12h and Supplementary Video 10).

To assess the role of WLS during brain organoid patterning, we generated a *WLS*-knockout (*WLS*-KO) iPS cell line using CRISPR–Cas9 genome editing (Extended Data Fig. 12i,j). Control and *WLS*-KO organoids were cultured with five different treatments (Matrigel, no extrinsic matrix, WNT activator (CHIR99021 pulse), YAP1 activator (Py-60 pulse) or DMSO control) and analysed at day 55 using scRNA-seq (Fig. 5h and Extended Data Fig. 12k). Integration and clustering of the scRNA-seq data revealed varying proportions of telencephalic progenitors and neurons, retinal progenitors, choroid plexus, neural crest cells, mesencephalic and rhombencephalic progenitors, and neurons between the different genetic and culture conditions (Fig. 5i,j). Specifically, *WLS*-KO organoids cultured without extrinsic matrix do not contain an increased proportion of non-telencephalic lineages, but instead show cell populations similar to control organoids grown with Matrigel (Fig. 5j). Both activation of WNT signalling and YAP1 in control organoids showed depletion of telencephalic cell fate and instead emergence of choroid plexus, retinal, mesencephalic and rhombencephalic progenitors; however, these cell composition changes were not observed in *WLS*-KO organoids (Fig. 5i–k). Genes upregulated upon WNT activation in control organoids included *WLS*, *EMX2*, *RSPO2* and *CRABP2*, whereas genes expressed higher in *WLS*-KO organoids included *FOXG1*, *SOX4*, *MEIS2* and *HES5* (Fig. 5k, Extended Data Fig. 12l and Supplementary Table 9). Genes upregulated upon YAP1 activation in control organoids included *WLS*, *MSX1*, *NR2F2* and *COL1A1*, whereas genes expressed higher in *WLS*-KO organoids included *FOXG1*, *SOX4*, *SIX3* and *SFRP1* (Fig. 5k, Extended Data Fig. 12m and Supplementary Table 10). Together, we have shown that in the absence of an extrinsic matrix, YAP1 is upregulated during early brain organoid development, which in turn induces expression of WLS, promoting caudalization of the developing neuroepithelium.

## Discussion

Understanding the developmental dynamics of human neural tube morphogenesis and patterning, corresponding to the first 2–5 weeks of human embryonic development, have remained obscure due to challenges in accessing live neural tissue. Brain organoids enable modelling important aspects of human neuroepithelium morphogenesis in vitro; however, technical challenges have hindered insight into the dynamics of how neural tissues self-organize, including the opaque nature of the tissue and long developmental times. Here we overcame these obstacles, harnessing the modularity inherent to brain organoid protocols to generate sparsely labelled, multi-mosaic organoids. This strategy enables incorporation of multiple fluorescent reporters that can be imaged at high spatiotemporal resolution for several days, leveraging the low phototoxicity offered by light-sheet microscopy^46,47^. Multi-mosaic and sparse labelling offers the advantage to multiplex, segment and measure single-cell dynamics from multiple reporter labels while also providing a buffer to cell-line-specific behaviours. The disadvantage is that sparse labelling affects lumen segmentations, cell lines are not homogeneously interspersed within the organoid and clonal expansion within regions limits multimodal readouts within one mosaic organoid, thus requiring sampling of several organoids. We achieved sterile growth conditions that enabled long-term tracking of cellular and subcellular signals at single-cell resolution, and developed a computational pipeline to track cells and identify structural morphotypes using this imaging modality. Our analysis pipeline streamlines pre-processing and post-processing of raw image data to generate segmented lumen and organoid masks to track tissue-level morphological changes, and to generate cell masks to allow tracking and quantification of cell-shape morphometrics and alignments from hundreds to thousands of cells spanning an entire week of organoid development. Using this system, we have generated a detailed characterization of the cell and tissue dynamics in unguided brain organoid development from induction of the neuroectoderm to formation of a patterned neuroepithelium that gives rise to distinct brain regional progenitors. The ECM and the mechanical environment have been postulated to have a role in both the morphogenesis and the patterning of the neural tube; however, the role of an externally provided matrix in neuroepithelium organization has remained unclear.

Using our long-term live-imaging framework in combination with single-cell transcriptomic analyses, we have demonstrated that the ECM provided on the outer margin of organoids induces stem cells to efficiently form the neuroectoderm with polarized cells that arrange perpendicular to expanding, ventricle-like lumens and eventually influences patterning of the tissue into largely telencephalic domains. In the absence of an extrinsic matrix, organoids showed mixed cell alignments without apicobasal polarity oriented in an inner–outer axis around the lumen, resulting in accumulation of the secreted ECM in both the lumen cavity and outside the organoid. We found that canonical WNT and YAP1–Hippo pathways are involved in this matrix-mediated neuroepithelium alteration, which has an influence on both rostrocaudal and dorsoventral brain organoid patterning. Ectopic YAP1 (ref. ^48^) activation also led to organoid caudalization with an increase in non-telencephalic cell types, which was associated with loss of lumen expansion. *WLS* and other WNT pathway-related genes were similarly modulated under no-matrix and YAP1 activation conditions, suggesting that YAP1 has a role during both matrix-mediated morphogenesis and organoid patterning. Loss-of-function experiments confirmed the role of WLS in brain organoid patterning and formation of non-telencephalic regions. Together, our work introduces a technological advance towards understanding the morphodynamics of organoid development, provides mechanistic insights into matrix-mediated neuroepithelial signalling pathways, and paves the way for future explorations of the extracellular microenvironment during human brain development.

## Methods

### Experimental methods

#### Stem cell and organoid culture

We used the following induced pluripotent stem (iPS) cell line for all experiments (also see Supplementary Methods Table 1): histone2B–mEGFP that uniformly labels nuclei (cell line ID: AICS-0061-036, cl.036), mEGFP–β-actin that uniformly labels ACTB (cell line ID: AICS-0016-184 cl.184), mTagRFP–T-CAAX that labels cell membrane (cell line ID: AICS-0054-091, cl.091), mTagRFP–T-tubulin-α1b that labels TUBA1B (cell line ID: AICS-0031-035, cl.035), mTagRFP–T-laminB1 that labels LMNB1 (cell line ID: AICS-0034-062, cl.062) and unlabelled WTC iPS cells (cell line ID GM25256). NKX2.1–GFP/w human embryonic stem cells were obtained from A. Kirkeby’s research group at the University of Copenhagen, after the arrangement of an MTA with E. Stanley and A. G. Elefanty (Murdoch Childrens Research Institute). Stem cell lines were cultured in mTSR^+^ (mTeSR Plus, StemCell Technologies) with mTSR^+^ supplement (StemCell Technologies) and supplemented with penicillin–streptomycin (pen-strep; 1:200; 15140122, Gibco) on Matrigel-coated plates (354277, Corning). Cells were passaged 1–2 times per week using TryplE (12605010, Gibco) or EDTA in DPBS (final concentration of 0.5 mM; 12605010, Gibco). The cell culture medium was supplemented with 1:1,000 Rho-associated protein kinase inhibitor (ROCKi) Y-27632 (final concentration of 5 μM; 72302, StemCell Technologies) on the first day after passage. All cell lines were tested for mycoplasma infection regularly using PCR validation (Venor GeM Classic, Minerva Biolabs) and found to be negative. The organoid generation protocol (multi-mosaic and sparse organoids) was as follows: 500 cells (except Extended Data Fig. 1b, which has 3,000 cells) in mTSR^+^ (with 1:200 ROCKi and 1:200 pen–strep) were added per well of a 96-well plate (CLS7007, Corning) and centrifuged at 200*g* for 5 min to generate embryoid bodies. Fresh mTSR^+^ with 1:200 ROCKi and 1:200 pen–strep was exchanged on day 2. Fresh NIM with 2% dissolved Matrigel was supplied on day 4 and exchanged every other day, followed by differentiation medium without vitamin A (VitA) and with 2% Matrigel on day 10 and differentiation medium with VitA and with 1% Matrigel on day 15. Organoids were moved to 24-well plates on day 15, one organoid per well, and moved to a shaker, followed by moving one organoid per well to a 6-well plate at 1 month and kept on a shaker. The no-matrix organoids were cultured following the exact same conditions, without any addition of Matrigel at any point. For agarose embedding, organoids were embedded in 0.6% or 0.3% low-melting agarose (SeaPlaque agarose, 501010); stock was 1%, in PBS, diluted to 0.6% in NIM. The use of human embryonic stem cells for the generation of brain organoids was approved by the Ethics Committee of Northwest and Central Switzerland (2019-01016) and the Swiss Federal Office of Public Health. The composition of NIM, differentiation medium without VitA and differentiation medium with VitA was based on earlier work^26^.

##### Neural induction medium

To make 250 ml of NIM, the following were combined: 250 ml DMEM/F12, 2.5 ml N2 supplement, 2.5 ml Glutamax supplement, 2.5 ml MEM-NEAA, 50 μl heparin solution (5 mg ml^−1^) and 1.25–2.5 ml pen–strep, 0.22-μm filtered and stored at 4 °C for up to 2 weeks.

##### Differentiation medium without vitamin A

To make 250 ml of differentiation medium without VitA, the following were combined: 125 ml DMEM/F12, 125 ml neurobasal, 1.25 ml N2 supplement, 2.5 ml B27 without VitA supplement, 62.5 μl insulin, 227.3 μl 55 mM 2-mercaptoethanol solution, 2.5 ml Glutamax supplement, 1.25 ml MEM-NEAA and 2.5 ml pen–strep, 0.22-μm filtered and stored at 4 °C for up to 2 weeks.

##### Differentiation medium with vitamin A

To make 1,000 ml of differentiation medium with VitA, the following were combined: 500 ml DMEM/F12, 500 ml neurobasal, 5 ml N2 supplement, 10 ml B27 + VitA supplement, 250 μl insulin, 909.2 μl 55 mM 2-mercaptoethanol, 10 ml Glutamax supplement, 5 ml MEM-NEAA and 10 ml pen–strep, 0.22-μm filtered and stored at 4 °C for up to 2 weeks.

For the organoid time-course scRNA-seq (Figs. 1 and 2), brain organoids were generated from the histone2B–mEGFP cell line (cell line ID: AICS-0061-036). Organoids from days 5–11 belonged to the same batch, days 16–21 to a different batch and day 30 to a third batch. Multiple organoids of each line were pooled together to obtain a sufficient number of cells. For the early time points (days 5, 7 and 11), 24 organoids, each grown in an independent well of a 96-well plate, were pooled, decreasing to 12 organoids for day 16, 10 organoids on day 21 and 6 organoids on day 30. For the day 13 scRNA-seq (Extended Data Fig. 10) with Matrigel (11 organoids), no-matrix (18 organoids) and agarose embedding (11 organoids), the organoids were generated from the unlabelled WTC parent iPS cell line. Agarose was degraded using cell recovery solution (11543560, Corning) at 4 °C. For the experiments with YAP activator (Fig. 5e), 16 control organoids, 23 organoids from Py-60 (given on day 5) and 20 organoids from Py-60 (given on day 7) were hashed together and used for scRNA-seq. For the experiments in Extended Data Figs. 11 and 12), 5 control organoids (Matrigel), 4 no-matrix organoids and 13 organoids from Py-60 treatment were hashed and used for scRNA-seq. For the experiments with control and *WLS*-KO organoids (Fig. 5), organoids were generated from the control and KO cell lines (see below), 12 control and *WLS*-KO organoids each from Matrigel and no-matrix conditions, and 8 control and *WLS*-KO organoids each from Matrigel condition (DMSO control, CHIR99021 and Py-60) were hashed together and used for scRNA-seq.

For scRNA-seq (Fig. 5e,f), sparse and multi-mosaic organoids containing cells labelled with nuclear membrane (lamin, RFP), actin (GFP) and tubulin (RFP) and unlabelled cells were cultured as described above with the addition of 1:1,000 DMSO in NIM to control organoids on day 5 and 10 µM Py-60 (HY-141644, MedChem Express) to organoids on day 5 or day 7. Media with fresh inhibitor or control media were exchanged every other day until dissociation and sequencing on day 10. For scRNA-seq (Extended Data Figs. 11c–f and 12c–g), embryoid bodies were generated using the unlabelled WTC-11 parent iPS cell line from 500 cells aggregated in mTSR^+^ (1:200 ROCKi and 1:200 pen–strep), per well of a 96-well plate, and centrifuged at 200*g* for 5 min to generate embryoid bodies. Fresh mTSR^+^ with 1:200 ROCKi and 1:200 pen–strep was exchanged on day 2 and day 4. Fresh NIM was supplied on day 6 and exchanged every other day, followed by differentiation medium without VitA on day 10 and differentiation medium with VitA on day 15. Organoids were given 2% Matrigel, 10 µM Py-60 with 2% Matrigel or no-matrix on day 10. All media were exchanged on day 13 followed by dissociation and sequencing on day 16. For the scRNA-seq in Fig. 5h–k, organoids were generated from *WLS*-KO and control cell lines with small modifications: 500 cells in mTSR^+^ (with 1:200 ROCKi and 1:200 pen–strep) were added per well of a 96-well plate (CLS7007, Corning) and centrifuged at 200*g* for 3 min to generate embryoid bodies. Fresh mTSR^+^ with 1:200 ROCKi and 1:200 pen–strep was exchanged on day 2. Fresh NIM with 2% dissolved Matrigel was supplied on day 4 (Matrigel condition) and exchanged every other day. The organoids were given differentiation medium without VitA with 1% dissolved Matrigel from day 10 to day 18 with fresh media exchanged every other day. On day 10, the Matrigel organoids were divided into Matrigel, Matrigel (DMSO control), Matrigel (CHIR99021) and Matrigel (Py-60) conditions. The organoids were treated with 1:1,000 DMSO, 2 µM CHIR99021 (2-day pulse) or 10 µm Py-60 (4-day pulse) in differentiation medium without VitA. CHIR99021 was washed on day 12, with fresh media exchanged, Py-60 and DMSO were refreshed in media exchanged on day 12 for an additional 2 days and washed on day 14. Organoids were given differentiation medium with VitA with 1% Matrigel on day 18 and moved to 24-well plates, one organoid per well, and were not moved to a shaker until use for scRNA-seq on day 55. No-matrix organoids were treated the same without any Matrigel addition and without Chiron or PY-60 treatments.

#### *WLS*-KO and control line generation

The human iPS cell line WTC-TUBA1BmTAgRFP-T (cell line ID: AICS-0031-035, cl.035) was used to create the *WLS*-KO line. Two guides were designed using the scoring system from CHOPCHOP^49^ and the IDT Custom Alt-R CRISPR–Cas9 guide RNA generator tool. The following two guides were selected to target the *WLS* gene: ACTCAGCAAACGCGTCATCACGG and ACGAGCGGAACCACATCGCAGGG. The Alt-R CRISPR–Cas9 System (IDT) was used for guide delivery with electroporation, using the Lonza 4D-Nucleofector X Unit with the 20 µl 16-well strips according to the manufacturer’s protocol. To form the CRISPR RNA (crRNA)–*trans*-activating crRNA (tracrRNA) complex in a final concentration of 2.3 µM for each guide complex, 0.6 µl of each guide crRNA was combined with 0.6 µl of tracrRNA in a separate tube. The 1.2 µl of each crRNA–tracrRNA complex was then combined with 1 µl of Alt-R S.p. HiFi Cas9 Nuclease V3 (10 µg µl^−1^; 1081060, IDT) and 0.3 µl of Duplex Buffer (IDT) in separate tubes. For electroporation, the two loaded RNP complexes were combined with 1 µl electroporation enhancer and added to 20 µl P3 (V4XP-3032, Lonza) containing 2 × 10^5^ cells. Cells were electroporated using the program H9. After electroporation, cells were incubated with 80 µl mTSR^+^ (100-0276, StemCell Technologies) with CloneR (final concentration of 1:1; 05888, StemCell Technologies) for 10 min in the 16-well strip and later split into 3 wells of a 12-well plate coated with Matrigel (35248, Corning) containing 0.5 ml mTSR^+^ (100-0276, StemCell Technologies) with CloneR (final concentration of 1:10; 05888, StemCell Technologies). After 24 h, the medium was replaced with 0.5 ml mTSR^+^ (100-0276, StemCell Technologies) supplemented with 1:200 pen–strep, and the cells were allowed to recover for 72 h. The cells were passaged onto CellAdhere Laminin-521 (77003, StemCell Technologies) coated well in StemFlex (A3349401, Gibco) with Y-27632 (final concentration of 10 µM; 72302, StemCell Technologies). Single clones were then generated using iotaSciences IsoCell according to the manufacturer’s protocol. Single-cell solution with 7,500 cells per millilitre was prepared for the IsoCell and two provided grids prepared. Singularity of clones was tracked using an EVOS XL Core over the course of 7 days. Single clones were harvested in StemFlex (A3349401, Gibco) with CloneR (final concentration of 1:10; 05888, StemCell Technologies) and distributed into a 96-well plate coated with Matrigel (35248, Corning). After 24 h, medium was exchanged to mTSR^+^ (100-0276, StemCell Technologies) supplemented with 1:200 pen–strep. After 48 h, clones were passaged to 12-well plates using 90% of the cell suspension, and 10% was used for validation of frameshift mutations by sequencing and analysed with CRISPResso2 (ref. ^50^). Clones were then cryopreserved. The control line and *WLS*-KO line used in this study were karyotyped and showed a normal karyotype.

#### Organoid dissociation and scRNA-seq

For all experiments, single-cell suspensions were generated by dissociation of the organoids with a papain-based neural dissociation kit (130-092-628, Miltenyi Biotec). In brief, organoids were washed three times with HBSS without Ca^2+^ and Mg^2+^ (37250, StemCell Technologies). Pre-warmed papain solution (1–2 ml) was added to the organoids and incubated for 15 min at 37 °C. The tissue pieces were triturated 5–10 times with 1,000 µl wide-bore and then P1,000 pipette tips. The tissue pieces were incubated twice for 10 min at 37 °C with additional trituration steps in between and after with P200 and P1,000 pipette tips. Cells were filtered consecutively with a 30-µm or 40-µm filter, centrifuged at 300*g* for 5 min and resuspended in cold PBS. The viability and cell count for the single-cell suspensions were assessed using a Trypan Blue assay on the automated cell counter Countess (Thermo Fisher Scientific). Cell suspensions from days 5, 7, 11, 16 and 21 were cryopreserved in Bambanker (BBH03, Nippon Genetics Europe) and stored at −20 °C until the scRNA-seq experiments were performed. The cryopreserved single-cell suspensions of each time point were thawed by warming up the cryo for 1–2 min in a water bath at 37 °C and directly centrifuged in 10 ml pre-warmed DMEM with 10% FBS. Cells were washed twice with PBS + 0.04% BSA and filtered through a 40-µm cell strainer (Flomi). For scRNA-seq, cells were resuspended to a final concentration after counting and viability checking that enabled targeting 8,000 cells and, in case the cell numbers were not sufficient, all cells were loaded. The scRNA-seq libraries were generated using the Chromium Single Cell 3′ V3 Library & Gel Bead Kit. Single-cell encapsulation and library preparation were performed according to the manufacturer’s protocol.

#### Cell hashing and scRNA-seq

For scRNA-seq (Fig. 5 and Extended Data Figs. 11 and 12), single-cell suspensions were obtained after organoid dissociation (described above) and cells of different samples were multiplexed using Cell Hashing^51^. Cell hashing was performed as described in the TotalSeq-A Antibodies and Cell Hashing with 10x Single Cell 3’ Reagent Kit v3.1 (Dual Index) protocol (https://www.biolegend.com/fr-ch/protocols/totalseq-a-dual-index-protocol). In brief, approximately 350,000 cells were resuspended in 45 μl DPBS + 0.5% BSA, 5 µl of Human TruStain FcX (Fc Receptor Blocking Solution, 422302, BioLegend) and cells were incubated for 10 min on ice. After blocking, 2 µl (1 µg) of each TotalSeq-A anti-human Hashtag antibodies (A0251-A065, BioLegend) were added per sample and were incubated for 30 min on ice with gentle agitation every 10 min. The cells were then washed twice with DPBS + 0.5% BSA and resuspended in 40 µl of DPBS + 0.5% BSA. Cells were counted and combined in equal ratios before processing them using Chromium Next GEM Single Cell 3′ Reagent Kits v3.1 (10x Genomics) according to the user guide (CG000206 Rev D, 10x Genomics). The library preparation of the HashTag Oligos was performed according to the TotalSeq-A Antibodies and Cell Hashing with 10x Single Cell 3’ Reagent Kit v3.1 (Dual Index) protocol. The libraries were sequenced according to the manufacturer’s guidelines on the Illumina NovaSeq platform.

#### Indirect iterative immunohistochemistry (4i)

The complete list of antibodies used for the 4i cycles is provided in Supplementary Methods Tables 2 and 11. For a detailed list of buffer compositions, see Supplementary Table 12. The 4i protocol was based on previous work^34,52^ and was as follows: organoids were fixed in 4% paraformaldehyde at 4 °C after harvesting, whereafter they were stored in 70% EtOH at −20 °C until use. Organoids were first embedded in a drop of 2% agarose due to their small size, then stored in 70% EtOH. The samples were then embedded in paraffin using a robot (TPC15, Medite) at 37 °C. For plate preparation, a Schott Nexterion 110 × 75 mm #1.5 glass plate was prepared in the following way: 10′ plasma asher for cleaning and surface activation, and immediately after, functionalization with poly-l-lysin (0.1 mg ml^−1^) with incubation for 1 h at room temperature. Next, it was rinsed twice with PBS, dried and stored dust free. Paraffin-embedded sections were cut at 3 µm, loaded onto the glass plate followed by drying at 40 °C overnight. They were then moved to 60 °C for 1 h and immediately placed in a Neoclear bath for de-paraffination. The plate was treated with 3′ NeoClear I, 3′ NeoClear II, 3′ 100% EtOH I, 3’ 100% EtOH II, 3′ 96% EtOH, 3′ 70% EtOH and 5′ ddH_2_O. This was followed by a 4% paraformaldehyde fixation (incubation for 15 min at room temperature) and rinsed with ddH_2_O. Aldehyde groups were blocked with 50 mM NH_4_Cl (incubation for 45 min at room temperature) and then the plate was rinsed with ddH_2_O. This was followed by heat-induced antigen retrieval with 10 mM citrate and 0.05% Tween-20 at pH 6.0. The plate was then heated up to 90 °C for 20 min with a histological microwave, then slowly cooled (in the microwave) to room temperature overnight. The plate was then fixed on a single-well frame with tape adhesive (Pentel tape ‘n glue) and sealed water tight with rubber cement. The samples were rinsed with 5% glycerol to prevent drying. The plate was then rinsed with ddH_2_O and PBS and stored with PBS at 4 °C until the start of the staining cycles. 4i cycles were then executed on a Felix Robot. Plate was rinsed three times with ddH_2_O and three times with 10 ml elution buffer for 10 min at room temperature at 300 rpm. It was washed three times with PBS. Blocking was done with 1.5% BSA, 0.1% Triton X100 and 0.1 M maleimide for 60 min at room temperature at 300 rpm. The plate was rinsed with PBS (to remove maleimide). Primary antibody hybridization was done in 1% BSA with 0.1% Triton X-100 for 45 min at room temperature at 300 rpm. The plate was washed three times with 1× PBS. Secondary antibody hybridization was done in 1% BSA and 0.1% Triton X-100 for 30 min at room temperature at 300 rpm. The plate was washed with PBS. The plate was rinsed two times with ddH_2_O followed by addition of imaging buffer.

#### Indirect iterative immunohistochemistry (4i) image acquisition

The 4i imaging was done on samples mounted on a large glass plate (110 × 75 mm) that was glued under a SBS-size superstructure. Imaging was performed on a Nikon Ti2 inverted microscope, coupled to a Crest X-Light V3, equipped with Teledyne Kinetix back-thinned cameras. The objective used was a Nikon Apochromat 40×/1.15 water immersion LWD (MRD77410) with water supply at 60 µl h^−1^ for 25 h. The following imaging conditions were used: DAPI at 80% power (100 ms), GFP at 80% power (300 ms), RFP at 80% power (300 ms) and Cy5 at 80% power (300 ms).

#### Light-sheet microscopy

All cell lines used for imaging were procured from the Coriell Institute as described above. For imaging, embryoid bodies were embedded in 20–50% Matrigel in neural-induction medium on day 4, one organoid per microwell and up to 16 organoids in one sample chamber. After gelification of Matrigel, NIM was added to the sample chamber and exchanged every other day. For the imaging experiments with ECM perturbations in Figs. 2 and 3, the sample mounting chamber has a vertical separation to segment it into 4 sub-chambers containing 4x organoids each. Embryoid bodies (4) were either embedded in 20–50% Matrigel dissolved in NIM, or 4 embryoid bodies were covered with 0.6% low melting agarose and for the no-matrix condition, 8 embryoid bodies were added to the sample chamber without any embedding. For the imaging shown in Extended Data Fig. 2d, embryoid bodies were aggregated from HES3 line (NKX2-1:GFP) using iPS Brew and rock inhibitor (1:200). After one day of aggregation, the embryoid bodies were transferred to a sample mounting chamber and embedded in Matrigel. A neural patterning medium (described in ref. ^53^) was employed for 14 days, complemented with SB432542 (Miltenyi, 130-106-543) and rh-Noggin (Miltenyi, 130-103-456) mediated dual SMAD inhibition from day 0 to day 9. After this, medium was changed to neural differentiation medium with VitA (composition previously described^26^) until day 21. The organoids were treated with the average morphogen concentration of SHH (140 ng/ml, Miltenyi, 130-095-727) from day 3–14 together with purmorphamine (0.21 µM, Miltenyi, 130-104-465). 3.5 µM XAV939 (Miltenyi, 130-106-539) was added from days 0–9. Medium was exchanged after every 2 days. Imaging was done with the LS1 Live light-sheet microscope developed by Viventis Microscopy, using a 25× objective demagnified to 18.5×, with a field of view that was approximately 710 µm and *xy* pixel size of 0.347 µm. Successive *z* steps were acquired every 2 µm for 201 steps. The frame rate for acquisition was 30 min for Fig. 1 and Extended Data Figs. 2e and 3a–c. The frame rate for acquisition was 60 min for Figs. 2, 3h–o and 5g and Extended Data Figs. 2f, 3, 4d-j, 5, 6e, 7 and 12h.

#### Fixation and whole-mount HCR

For whole-mount staining, organoids were fixed overnight at 4 °C on the nutator, washed 3–5 times in PBST, dehydrated with a PBST–methanol gradient (50% and 100%) and stored at −20 °C in 100% methanol until use. All probe sets were designed and provided by Molecular Instruments. The amplifiers and buffers were also ordered from Molecular Instruments (https://www.molecularinstruments.com/). HCR was performed according to the manufacturer’s protocol provided by Molecular Instruments with small changes. All five hairpins (B1–B5) conjugated with the following dyes were used per experiment (Alexa-488, Alexa-514, Alexa-545, Alexa-594 and Alexa-639). In brief, the samples were rehydrated with a series of graded methanol–PBST washes (25%, 50%, 75% and 100%) for 5 min each at 4 °C on the nutator and washed an additional time with PBST. The samples were then treated with 10 µg ml^−1^ proteinase K (25530-049, Invitrogen) for approximately 3–5 min at room temperature followed by two times 2× PBST washes for 5 min. They were then post-fixed with 4% paraformaldehyde for 20 min at room temperature and washed three times with PBST for 5 min each. The organoids were pre-hybridized in the probe hybridization buffer for 30 min at 37 °C. Of each probe set, 1 pmol was diluted into probe hybridization buffer and the samples were incubated overnight at 37 °C. The next day, the samples were washed four times with the probe wash buffer at 37 °C and washed two more times with 5× SSCT. The organoids were then incubated in the amplification buffer for 10 min at room temperature followed by adding snap-cooled hairpin mixture diluted in the amplification buffer to incubate overnight at 25 °C. The excess hairpins were washed the next day with 2 × 5 min washes as well as two longer washes of 30 min followed by 1 × 5 min wash with 5× SSCT buffer at room temperature. Organoids were stained with DAPI (1 µg µl^−1^) during the first 30 min washes. The samples were stored at 4 °C and mounted on a µ-Slide chamber (80807, Ibidi) and covered with 1% agarose. The samples were imaged using a ×10 water immersion and 0.8 NA objective on the Zeiss LSM980 Airyscan system. Images were acquired using lambda scanning followed by spectral unmixing to image 6 channels in three sets of excitation (first round 514 nm + 639 nm, 2nd round 488 nm + 545 nm + 594 nm, and 3rd round 405 nm). All images were processed using Fiji and the BigDataViewer plugin^54,55^.

#### Bulk CUT&Tag for YAP1

Organoids were generated from the WTC-11 iPS cell line by culturing embryoid bodies (as described above) for 6 days, following which NIM was added on day 6. On day 10, differentiation medium without VitA was given with 2% dissolved Matrigel. Single-cell suspensions of 12-day-old organoids were prepared using the Miltenyi Neural Tissue Dissociation Kit (P) (130-092-628) following the manufacturer’s guidelines. Cells were counted and directly transferred into CUT&Tag Wash buffer supplemented with 0.01% digitonin (20 mM HEPES pH 7.5, 150 mM NaCl, 0.5 mM spermidine and 1× Roche protease inhibitor cocktail). Per experiment, 1 million cells were used and incubated with 2 µg YAP antibodies (ab52771, Abcam and sc-101199, Santa Cruz). All following steps were performed as previously described^56,57^, except that the Tn5 incubation and cutting were performed at a NaCl concentration of 150 mM. To control unspecific cutting, we performed the experiment without antibody (Tn5 only) and with a generic anti-rabbit antibody. The proteinA-Tn5 was purified in house as previously described^56^. Final libraries were sequenced on the NovaSeq platform with PE 2 × 50-bp read length. Sequenced reads were mapped against hg38 using Bowtie2 (ref. ^58^). These were filtered for PCR duplicates and mapping quality. Coverage tracks were then generated using deeptools2 bamCoverage and normalized by sequencing depth^59^. Tracks were visualized using IGV.

#### RT–qPCR for YAP1 perturbation screen

Organoids were generated from the WTC-11 iPS cell line as described above and cultured without Matrigel. Organoids were treated with 1:1,000 DMSO (control), YAP1 activators (10 μM TRULI, 10 μM GA-017 and 10 μM Py-60) or inhibitor (10 μM TED-34) in differentiation medium without VitA on day 10 and refreshed on day 13. Eight organoids per condition were harvested for qPCR in TRIzol and used for RNA extraction. Of each sample, 50 ng RNA was reversely transcribed into cDNA using the ReadyScript cDNA Synthesis Mix (RDRT, Sigma-Aldrich), with the following cycler programme: 5 min at 25 °C, 30 min at 42 °C, 5 min at 85 °C and hold at 4 °C in a C1000 Thermal Cycler (Bio-Rad). RT–qPCR was then performed for *WLS* gene expression quantification and GAPDH (used as an internal normalization control). The WLS primers were forward-gccagctatgagcaaagtcc and reverse-tgggatggtgcatacaagaa. The GAPDH primers were forward-GGAGCCAAACGGGTCATCATCTC and reverse-GAGGGGCCATCCACAGTCTTCT.

RT–qPCR was performed using KAPA SYBR FAST qPCR Kit (KK4602, Kapa Biosystems) according to the manufacturer’s instructions with the following cycler programme: 3 min at 95 °C; 45 cycles of 3 s at 95 °C, 20 s at 60 °C and 10 s at 72 °C; 10 s at 95 °C; 1 min at 65 °C; and 1 s at 97 °C in a LightCycler96 (Roche).

#### Synthetic PEG hydrogel

Eight-armed poly(ethylene glycol) (PEG) with vinyl sulfone (VS; 8-PEG-VS) end group (hexaglycerol octa(vinylsulfonylethyl) polyoxyethylene) was purchased from NOF (40 kDa). Eight-armed PEG with thiol functionality containing sortase-sensitive peptide sequence (8-PEG-SS-SH) was synthesized following a previous protocol^60^. The effective functionality was checked by ^1^H-NMR and Ellman assay for thiol concentration. The thiol-containing peptides used in this study, GFOGER (GGYGGGPG(GPP)_5_GFOGER(GPP)_5_GPC), scrambled control peptide RDG (GRCGRDGSPG) and dithiol SrtA sensitive (DSH-SS; GCRELPRTGERCG) were purchased from Biomatik. The hydrogel (1.75% w/v PEG content to reach elastic modulus of approximately 100 Pa) was formed by reacting 8-PEG-VS with 8-PEG-SS-SH and adhesion peptide (1 mM). For some conditions, laminin-111 (Trevigen) was incorporated into the final gel mixture at 0.6 mg ml^−1^. The organoids were embedded in the hydrogel mixture on a light-sheet membrane and left to polymerize for 30 min at 37 °C. After gelation, the culture media were then added to the hydrogel.

#### Sortase A production and gel degradation

Sortase A (SrtA; plasmid #75144, Addgene) with a 6×-His tag was expressed in *E. coli* BL21(DE3) following a published protocol^61^. The sortase degradation solution was prepared following a previous protocol^60^. In brief, a mixture of SrtA (15 µM) and triglycine (GGG, 200 mM; Sigma) was added to the NIM (day 8) and differentiation medium without VitA (day 13) samples. The degradation solution was added to the PEG hydrogels and left to degrade for 30–60 min at 37 °C until complete dissolution. The organoids were collected for further analysis.

#### Bulk RNA-seq analysis

Organoids frozen in 50 μl TRIzol Reagent (15596018, Thermo Fisher Scientific) were rapidly shaken until the tissue was completely dissolved. The volume of TRIzol was adjusted to 500 μl and total RNA was extracted with 100 μl of chloroform by rapid shaking for 15 s and subsequently centrifugation at 12,000*g* at 4 °C for 15 min. The resulting aqueous phase was re-extracted with an equal chloroform volume. Following centrifugation, the aqueous phase was mixed with an equal volume of isopropanol and RNA was precipitated along with the GlycoBlue coprecipitant (AM9516, Thermo Fisher Scientific) for an average of 4 days at −20 °C. Subsequently, the samples were centrifuged at 12,000*g* at 4 °C for 30 min. The resulting RNA pellets were washed two times with ice-cold 80% ethanol, then dried on ice for 10 min and resuspended in 20 μl of the TURBO DNA-free (AM1907, Thermo Fisher Scientific) mix. Following DNA digestion for 30 min at 37 °C and DNase inactivation according to the manufacturer’s guidelines, the dissolved RNA was centrifuged at 10,000*g* for 1.5 min at 4 °C. RNA aliquots were immediately frozen on dry ice and subsequently stored at −80 °C. RNA traces and concentrations were examined using the Qubit RNA BR Assay kit (Q10211, Thermo Fisher Scientific) and RNA 6000 Pico Kit (5067-1513, Agilent Technologies). RNA dilutions for normalization purposes were prepared using RNase-free water in a 96-well plate. The cDNA was prepared and amplified according to the Smart-seq2 protocol^62^. An average of 10–15 ng of RNA (1 µl) was used as input into the reaction. The cDNA was purified using the SPRIselect reagent (B23318, Beckman Coulter) at the ratio of 0.8×, and the resulting cDNA traces and concentrations were examined using the Bioanalyzer DNA High Sensitivity kit (5067-4626, Agilent Technologies) and Qubit dsDNA High Sensitivity kit (Q32854, Thermo Fisher Scientific). Of normalized cDNA, 1.25  μl was used to construct Nextera libraries. The single organoid bulk RNA-seq libraries were subsequently pooled (3 µl each) and purified two times using the SPRIselect reagent (B23318, Beckman Coulter) at 0.9× ratio. The libraries were sequenced according to the manufacturer’s guidelines on the Illumina NovaSeq platform.

### Data analysis methods

#### Preprocessing of scRNA-seq data from the organoid time course

We used Cell Ranger (v3.0.2) with the default parameters to obtain transcript count matrices by aligning the sequencing reads to the human genome and transcriptome (hg38, provided by 10x Genomics, v3.0.0). Count matrices were further preprocessed using the Seurat R package (v4.3.0) and R (version 4.4.0)^63^. First, cells were filtered on the basis of the number of detected genes and the fraction of mitochondrial genes. As sequencing depth varied between time points, the threshold of the number of detected genes was set individually for each sample. For the scRNA-seq time course, the number of detected genes was between 1,000 and 7,500 and the mitochondrial genes threshold was less than 10%. Three thousand variable features were used and the number of PCA was set to 50. For Fig. 1b–d, the total number of cells per day after preprocessing were day 5 = 5,481, day 7 = 8,183, day 7 = 4,912, day 16 = 6,571, day 21 = 7,962 and day 30 = 7,950.

#### Integration of different time points

Integration of time points was performed using the log-normalized gene expression data of meta cells. We used the union of the selected genes and transcription factors and further excluded cell-cycle-related genes from the set. Next, we computed cell-cycle scores using the Seurat function CellCycleScoring(). Subsequently, the data were *z*-scaled, cell-cycle scores were regressed out (ScaleData()) and principal component analysis (PCA) was performed using the Seurat function RunPCA(). We used the first ten principal components (PCs) to integrate the different time points in the dataset using the cluster similarity spectrum (CSS) method. To remove any remaining cell-cycle signal for any downstream tasks, we again regressed out the cell-cycle scores from the integrated CSS matrix. To obtain a 2D representation of the data, we performed UMAP^64^ embedding using RunUMAP() with spread = 1.0, min.dist = 0.4 and otherwise the default parameters.

#### Pseudotime analysis of the early progenitor transition trajectory

Cells from day 5, day 7 and day 11, which were annotated as neuroectodermal (both neuroectoderm and late neuroecotoderm) were subsetted. CSS was used to re-integrate cells from different time points, and PCA was performed on the CSS representation to further reduce embedding dimensions to 10. A linear model was fit for each PCA-reduced CSS dimension, each with the cell-cycle scores as independent variables. Residuals of the linear models were obtained as the cell-cycle-regressed-out PCA-reduced CSS representation, which was then used as the input of the diffusion map (implemented in the destiny R package). The first diffusion component (DC1) was ranked across cells in the subset, and the ranked DC1 was used as the pseudotime representing the early progenitor transition progression.

#### Identification of genes with expression changed during early progenitor transition

For each gene detected in more than 1% of cells during early progenitor transition, two linear models were fit. The full model includes the natural spline (degree = 5) of pseudotimes, whereas the reduced model only contains intercept. Two tests were performed to compare the two models. The relaxed test used *F*-test to compare regression mean squares of the two models. The stringent test used *F*-test to compare mean square errors of the two models. To correct for multiple testing, the Bonferroni method was applied to the relaxed tests, and the Benjamini–Hochberg method was applied to the stringent tests. Genes with adjusted *P* < 0.05 in relaxed tests were defined as the broad set of genes with expression changed during the transition, whereas those with adjusted *P* < 0.01 in stringent tests were defined as the stringent set.

#### Preprocessing of scRNA-seq data from all other datasets

We used Cell Ranger (v3.0.2) with the default parameters to obtain transcript count matrices by aligning the sequencing reads to the human genome and transcriptome (hg38, provided by 10x Genomics, v3.0.0). Count matrices were further preprocessed using the Seurat R package (v4.3.0). The cells were filtered on the basis of the number of detected genes and the fraction of mitochondrial genes. As sequencing depth varied between time points, the threshold of the number of detected genes was set individually for each sample. For the matrix perturbation dataset (Extended Data Fig. 10), the number of detected genes was between 500 and 8,000, 3,000 variable features were used and the number of PCA was set to 50. Mitochondrial and histone-related genes were removed followed by integration. The total number of cells per condition after pre-processing were Matrigel = 8,095, no-matrix = 8,343 and agarose = 3,725. To obtain a 2D representation of the data, we performed UMAP embedding using RunUMAP() with the default parameters. For the YAP perturbation (Fig. 5), the number of detected genes was more than 200 and the mitochondrial genes threshold was set to less than 20%. Three thousand variable features were used and the number of PCA was set to 50. Mitochondrial, ribosomal and histone-related genes were removed followed by integration. The total number of cells per condition after preprocessing was control = 2,085, Py-60 given on day 5 = 763 and Py-60 given on day 7 = 1,955. To obtain a 2D representation of the data, we performed UMAP embedding using RunUMAP() with spread = 0.3 and min.dist = 0.5. For the *WLS*-KO scRNA-seq dataset in Fig. 5h–k, the number of detected genes was more than 500 and percent.mt of less than 10. Mitochondrial and ribosomal genes were removed followed by integration. Three thousand variable features were used and the number of PCA was set to 20. The total number of cells detected per condition after demultiplexing and preprocessing were Matrigel (wild type (WT)) = 3,560, Matrigel (*WLS* KO) = 1,661, no-matrix (WT) = 1,283, no-matrix (*WLS* KO) = 4,094, Chiron (*WLS* KO) = 1,774, Py-60 (*WLS* KO) = 1,383, Chiron (WT) = 3,310, Py-60 (WT) = 1,399, DMSO control (WT) = 2,087 and DMSO control (*WLS* KO) = 1,453. To obtain a 2D of the data, we performed UMAP embedding using RunUMAP() with spread = 0.6 and min.dist = 0.4. For the scRNA-seq datasets in Extended Data Figs. 11 and 12, the number of detected genes was more than 1,000 and percent.mt of less than 10. Mitochondrial, ribosomal and histone-related genes were removed followed by integration. Three thousand variable features were used and the number of PCA was set to 50. The total number of cells per condition after preprocessing were Matrigel = 1,183, no-matrix = 1,285 and YAP activator Py-60 = 1,189). To obtain a 2D representation of the data, we performed UMAP embedding using RunUMAP() with the default parameters. For all datasets, transcript counts were normalized to the total number of counts for that cell, multiplied by a scaling factor of 10,000 and subsequently natural-log transformed (NormalizeData()). The datasets in different experiments were integrated using CSS^65^.

#### Bulk RNA-seq analysis

Bulk RNA-seq reads were mapped to GRCh38 human genome using STAR (v2.7.11b). For each sample, the transcripts per million (TPM) were calculated for genes annotated in Ensembl v93 (filtered using the build steps in Cell Ranger Human reference 3.0.0, GRCh38) using RSEM (v1.2.28, rsem-calculate-expression). For each highly variable gene, a linear model was fit, with log-transformed TPM in Matrigel and no-ECM samples as the response variable, sample age and ECM condition as the independent variables. The coefficient of the ECM condition was effectively the approximation of overall log-transformed fold change (logFC) of gene expression in Matrigel in relative to no ECM. Next, the similar coefficients were calculated for the same genes, but for samples with the PEG-RDG condition and one of the PEG-GFO, PEG-LAM and PEG-LAM-GFO condition, as the estimation of transcriptomic effect by the addition to PEG. Spearman correlation coefficient was then calculated between Matrigel-to-no-ECM logFC and each of PEG-GFO, PEG-LAM and PEG-LAM-GFO with PEG-RDG, across the highly variable genes. The correlation quantifies how well the transcriptomic effect by Matrigel was recapitulated by the addition of PEG.

For each expressed gene, three linear models were fit, all of which use the log-transformed TPM as the response variable and sample age plus ECM condition as the independent variables. All the three models were fit using samples with different PEG media, but using samples at both day 8 and day 13, only day 8 and only day 13. ANOVA analysis of variance) was applied to each of the model for the *F*-test-based *P* value of ECM conditions. Benjamini–Hochberg multiple test correction was applied to tests using the same sample set. Genes with adjusted *P* < 0.05 in at least one of the three tests were considered as differentially expressed genes (DEGs) in PEG conditions. For each of the DEGs, logFC between each PEG conditions with PEG-RDG, as well as between Matrigel and no-ECM samples at each time point was calculated.

For each gene detected in more than half of the samples or average TPM across all samples larger than one (defined as expressed genes), the mean and coefficient of variation of expression were calculated. A generalized linear model with gamma distribution was fit between coefficient of variation and 1/mean for highly expressed genes among those with coefficient of variation > 0.3 (>median). Given the model, a Chi-square test was applied to each gene. Benjamini–Hochberg multiple test correction was applied to all the tested genes. Genes with the adjusted *P* < 0.05 were considered as highly variable genes.

### Light-sheet image analysis

#### Denoising and background subtraction

To increase the comparability between images in the dataset, all movies were denoised using Noise2void^66^ (v0.3.1). A separate 2D denoiser was trained for every position and every channel. The denoiser was trained on randomly selected *z* slices from throughout each corresponding movie and channel. For the datasets used in Figs. 1e–l and 3b,c,f,g and Extended Data Fig. 2e, 30 randomly selected *z* slices were used, and for the dataset in Figs. 2g–j and 3d,h–o and Extended Data Fig. 4d–j, 90 random *z* slices were used. The randomly selected *z* slices were split into a training subset with 90% of the *z* slices and a test subset with 10% of the *z* slices. The denoiser was trained for 128 epochs on the training subset with 96 × 96 pixel patches, with a batch size of 256, a unet_kern_size of 3, train_loss equal to mse and n2v_perc_pix of 0.198. The training progress was monitored on the test subset. The trained denoiser was then used to denoise all images in the movies. After this, the background subtraction was performed by using the minimal value along the *z* axis and clipping small intensity values. The Extended Data Fig. 2e dataset was further dehazed using the dehazing function from dexp^67^ with a filter size of 60 and correct_max_level set to false. For Fig. 1g, the 3D images were bi-linearnly rescaled to an isotropic voxel size of 1.15 μm. Then, a wedge mask was created with a triangular cutout and then multiplied with the downscaled image. The image was rotated using Scipy^68^ (v1.7.3) ndi.rotate function. The images were then attenuated as implemented in the dexp.processing.color.projection function with the parameters attenuation of 0.0005, attenuation_min_density of 0.3 and attenuation_filtering of 24. For the spinning movie, in Supplementary Video 2, the 3D image was bi-linearnly rescaled, with anti-aliasing, to an isotropic voxel size of 1.388 μm and 32 zero-padding planes were added to before and after in *x*, *y* and *z* directions. The image was then rotated with the scipy.ndi rotate function, and after each rotation, each channel was attenuated with the parameters attenuation_filtering = 4, attenuation_min_density = 0.002 and attenuation = 0.01.

#### Temporal registration

The light-sheet dataset from Fig. 1e–l and Fig. 3b,c,f,g was registered as follows. First, the movies were cropped and centred using the cropping algorithm implemented in LStree (v0.1)^27^. As only one organoid is present per position, the largest object was linked throughout the movie for cropping. After cropping, the images were padded with zeros, 32 planes before and after the image in the *z* direction, and with 96 planes before and after the image in both *x* and *y* direction before registration. For the temporal image registration, translational registration from itk-elastixs (v5.2.1)^69,70^ with the default translation parameter map was used to register the movies using GFP as the leading channel. The first frame in the movie was used as a reference frame and then the second image was registered to it. After this, the translation was applied to both channels of the second image. Then, the next image was registered to the previously registered frame in the movie, and again the translation was applied to both channels in the next image. This was repeated until all images in a movie were registered. After translation, the images were clipped to ensure that no small negative values remained. Movies in the positions 9–16 contained some higher-intensity areas. To deal with this, before calculating the registration, the image intensities were rescaled to the 1st and 99th percentile, and the default translation parameter map with three resolutions was used for registration. The calculated translation was then applied to both non-rescaled images for these positions.

Before quantifications and tracking, the light-sheet movies used in Figs. 2 and 3d were linearly downscaled to half resolution and a multi-frame comparison registration approach was used. The mean phase cross-correlation of the maximum intensity projection of the GFP channel across each axis was used to calculate the translation in *x*, *y* and *z* between any pair of images. This approach was used to calculate the translations across three consecutive time points for every frame in the movie. On the basis of this multiframe translation matrix, the averaged translation for each time point was calculated. The translations were then rounded to the next integer, and zero padding was calculated for the whole movie. After this, the translations were applied to all channels and masks by shifting the image along each axis and adding padding to prevent data loss.

#### Tissue segmentation

A pixel-wise random forest classifier was used for the tissue segmentation similar to other already established tools such as Weka segmentation^71^. Owing to the multi-terabyte size of the dataset, a Python pixel-wise segmentation classifier using both scikit-learn (v0.18.3)^72^ and scikit-image (v1.1.1)^73^ was implemented (https://scikit-image.org/docs/stable/auto_examples/segmentation/plot_trainable_segmentation.html#id4). The input for the random forest classifier were 2D image features from the sum of both channels, which were extracted using multiscale basic features function from scikit-image with a minimum sigma of 1, a maximum sigma of 64, and intensity, edge and texture features. The input image was bi-linearly downscaled to one-quarter resolution in *xy*. To train the classifier, random *z* slices from throughout the movies were annotated with bounding boxes in jupyter notebook using bboxwidgets (v0.4.0), as either background, lumen or organoid tissue (https://github.com/gereleth/jupyter-bbox-widget). Random slices were iteratively annotated until satisfactory image segmentations were produced. In total, 1,414 slices were corrected or annotated for the dataset used in Fig. 1j–l and 2,221 slices for the dataset used in Figs. 2h–j and 3n,o. For every slice with a bounding box, the 2D image features were extracted and a random forest classifier was trained to predict the labels. One classifier was trained for each dataset. The random forest classifiers for the two datasets were trained to a depth of 25, with 50 random trees and max_samples of 0.05. The trained models were then used to predict the labels for each pixel for each *z* slice in each time point of the movies. For both datasets, the segmentation masks were predicted for one time point per hour. After predicting the labels for each 2D segmentation, the output masks were stacked to a 3D segmentation mask. After this, the organoid and the lumen masks were extracted and smoothed by applying a scikit-images Gaussian filter with a voxel size of 1.388 in the *z* direction and 2 in both the *x* and *y* directions to the binary mask and then rebinarizing and combining the masks back to one segmentation image. After this step, each *z* slice of the 3D segmentation masks were further processed in 2D. First, small lumens were removed using the binary_closing function from scikit-image with a disk size of 3. Any holes in the lumen masks were filled and any background pixels within the organoid were assigned the lumen label. Furthermore, any voxels labelled lumen outside of the convex hull of the organoid were assigned the background label. The slice-wise 2D segmentations were then reassembled into a 3D tissue segmentation. As only one organoid is present in one movie, label and regionprops functions from scikit-image were used to extract the largest non-background object for further downstream analysis. Before tracking and morphology quantifications in Fig. 2h–j and Extended Data Fig. 4e–j, the lumen masks were upscaled to a 0.694 μm pixel size in *xy*, and two additional preprocessing steps were introduced to ensure temporal stability and reduce the amount of false-positive fusion detections. A temporal median filter was applied across all lumen masks of one movie with a filter size of 5 across the temporal axis and a size of 1 across the image directions (*xyz*). To reduce the number of false-positive fusions, a binary opening with four iterations was applied to the lumen masks. Finally, connected components were used to label the lumen and any lumen smaller than 20,000 μm^3^ was removed.

The resulting masks were visualized in 3D for Figs. 1i and 2h, Extended Data Fig. 4b,d,i,j, Supplementary Videos 4 and 8. The masks were rescaled to an isotropic resolution of 1.388 (Fig. 1i and Extended Data Fig. 4j) or 2.776 μm (Fig. 2h), extracting each lumen surface using scikit-images marching cubes, with a step size of 1 and level 0 and the organoid surface using marching cubes with a step size of 2 and level 0. Next, the surface meshes were cleaned using pymeshfix (v0.16.1; https://github.com/pyvista/pymeshfix/tree/main) clean_from_arrays and then meshes were smoothed using the filter_taubin function from trimesh (v3.13.0) (https://github.com/mikedh/trimesh) with 50 iterations. The surface meshes were then plotted using matplotlib (v3.5.2) plot_trisurf with the bounding box being equal to the maximum length in any of the *xy* and *z* direction. For Extended Data Fig. 4a and Supplementary Videos 3 and 7, using the organoid masks, the 90th percentile largest organoid *z* slice was calculated for every time point in every movie. Then, a sliding average was applied over the calculated 90th percentile *z* slices with a window size of 20 for Extended Data Fig. 4a and Supplementary Video 3 and a window size of 5 for Supplementary Video 7. Finally, for each of these slices, the images were false coloured according to the masks.

#### Tissue quantification

To characterize the tissue architecture, the organoid tissue and lumen sizes were quantified for the first 124 h for both Figs. 1j–l and 2h–j and Extended Data Fig. 4. For lumen quantification, any lumen with a size smaller than 20,000 was removed using scikit-images remove_small_objects function. Furthermore, scikit-images label and regionprops functions were used to assess the number of lumen, the major axis length and the size of each lumen. For Fig. 1j–l and Extended Data Fig. 4c, for the organoid at position 2, the measurements were not considered after hour 49, as the camera moved to another position. The organoids at position 4 between hours 43 and 48 were not considered as the organoids move out of the field of view. For Extended Data Fig. 3, the light-sheet movies were segmented using Ilastik and post-processed, as above, and scikit-images regionprops functions were used to assess the volumes and the major axis length.

#### Single-cell segmentation

To quantify changes in cell morphologies, 3D cell segmentation was performed using EmbedSeg^32^. One 3D image for every 24 h was created and was segmented for each movie. For the dataset in Fig. 3b,c,f,g, one position was segmented; for Fig. 3h–o, three positions per condition were segmented. The agarose condition was further supplemented with one additional red channel movie. Before training the model, the images were bi-linearly downscaled by a factor of 2 in the *xy* direction. Hand-selected 3D volumes were annotated using labkit^74^ and then used to train the model in a human-in-the-loop manner, where iteratively predicted 3D volumes were corrected and then used to retrain the model. In total, 5,850 cells were hand corrected in this manner. For creating the crops for training the model, the medoid was used and a n-sigma of 4 was used to calculate the crop size. Furthermore, a speed up factor of three was used to create the crops. The model tile size was set to 600 for both the *x* and *y* directions and 80 in the *z* direction. The model was trained on 5,512 cells, and 338 cells were set aside as a validation set. The 3D model was trained for 200 epochs, with a batch size of 8, and then the model that had the best iou performance of 0.72 on the validation set was selected. Using tiling, the 3D volumes were segmented, with a fg_thresh of 0.4, seed_thresh of 0.7 and test-time-augmentation set to true.

#### Cell morphology quantification

Morphometrics^75^ (https://github.com/morphometrics/morphometrics) was used to extract cell morphology measurements from the segmented images. The images were rescaled bi-linearly, and the masks with a nearest neighbour interpolation set to an isotropic voxel size of 0.694 µm. To speed up the measurements, masks with a volume smaller than 100 voxels (33.4 µm^3^) were removed using remove_small_objects from scikit-image. The surface, size, intensity, position and moment measurements were extracted for each cell mask using morphometrics. In addition, the major and the minor axis lengths for each cell were measured using the scikit-images regionprops function.

#### Quality control and demultiplexing

Before the manual quality control steps, masks with a volume of lower than 100 and a maximum intensity of less than 20 were removed from further analysis, leading to a total of 299,027 subcellular structures. Furthermore, any measurements containing NaNs (‘not a number’ values) were also removed from the analysis. Scanpy^76^ and morphometrics were used to normalize, dimensionally reduce using PCA and cluster (Leiden) the dataset. Then, random cells were extracted, from different time points, positions and clusters, and labelled to either be a histone, lamin, actin, tubulin, a cell membrane or a faulty mask. In total, 3,379 cells were hand annotated in this manner. The annotated cells were then split into a train (90%) and a test set (10%). For demultiplexing all measured measurements from morphometrics, channel information, as well as the major and minor axes were used. The training data were used to perform a fourfold cross-validation grid search on random forest classifiers from scikit-learn on the number estimators and the maximum depth of the tree. The best-performing random forest classifier was selected for demultiplexing and quality control. After this, wrongly demultiplexed structures, which either did not exist in the experiment or originated from the wrong channel, were removed. After manual quality control, a total of 59,053 structures remained. The model performance was then evaluated and achieved an accuracy of 0.73 on the test set. Furthermore, stratified fivefold cross-validation, across the whole dataset, was used to estimate the cross marker mispredictions. To do this, the model was retrained on the training set on each fold, the confusion matrix was calculated for each test set of a fold and then summed over all folds. Any low-quality mask predictions or labels were then removed, and the remaining protein markers were used to calculate the marker specific precision.

#### Morphology analysis

All morphological analyses were run using non-intensity-dependent measurements and additionally the axis length ratio was added. Scanpy^76^ was used to morphologically analyse the segment markers. For Fig. 3h–o, only actin cells from the ECM perturbation experiment were used and the measurements were scaled and dimensionally reduced using the pca function from morphometrics. The neighbourhood graph was then calculated using the first five principal components and then clustered using Leiden clustering with a resolution of 0.6 using the cluster features function from morphometrics. After this, clusters 8 and 9 contained only debris and were removed from the further analysis, and the data were rescaled, dimensionally reduced and clustered with the same parameters. After clustering, a graph embedding was calculated using PAGA^33^ with a threshold of 0.1, and the daily proportions of the different clusters for the conditions were calculated. For Fig. 3g,h, to obtain a 2D representation of the data, a PAGA-initialized UMAP embedding was calculated. On the basis of the axis length ratio of the cells in the clusters, the clusters were either coloured as containing elongated (cyan) or non-elongated cells (yellow). To create Fig. 3k, the first non-zero element from the 3D actin masks used in Fig. 3h–j was extracted. This was then used to extract a 2D image with the corresponding voxels from the intensity image and to colour the pixels according to the cluster membership. To estimate the diversity of the cell morphologies throughout the time series, the Shannon index was calculated for every time point and every condition: *H*′ = –∑_*i*_ *p*_*i*_ × log_2_(*p*_*i*_), *p*_*i*_ = *n*_*i*_/*N*, where *N* is the total number of cells of all clusters per day and condition and *n*_i_ is the number of members of a cluster for a specific condition and day. The confidence intervals were estimated through a bootstrap analysis, by randomly sampling with replacement from the whole dataset and recalculating the Shannon index for each condition and day. By sampling for 1,000 times, the 95% confidence interval was estimated. For Fig. 3b,c,f,g, all demultiplexed markers were used and the neighbourhood graph was then calculated using the first 4 principal components and then clustered using Leiden clustering with a resolution of 1.5. After clustering, a graph embedding was calculated using PAGA^33^ with a threshold of 0.1 and a PAGA-initialized UMAP embedding was calculated. For the PAGA path heatmaps, the diffusion pseudotime starting at cluster 2 was calculated and then plotted with an n-average of 50. For Extended Data Figs. 6e and 7b, the neighbourhood graphs were then calculated using the first 4 principal components and then clustered using Leiden clustering with a resolution of 0.4. After this, the PAGA graphs and the PAGA-initialized UMAPs were calculated. The PAGA cluster analysis was repeated using a Leiden clustering resolution of 16 and PAGA plots and heatmaps were created for the Extended Data Fig. 7e,f with an *n*-average of 20.

#### Cell angle analysis to the surface

To calculate the cell angle to the surface, first an ellipsoid was used to fit to the surface of the 3D masks of the individual cells (https://github.com/aleksandrbazhin/ellipsoid_fit_python). The surfaces of the binary subcellular feature masks were extracted using the marching_cubes function from scikit-image with a step size of 2 and level 0. Then, to estimate the directionality of the cell masks, the major axis of the 3D ellipsoid was extracted. Any cells where the ellipsoid had negative radii or radii larger than 180 voxels were excluded from further analysis. The organoid mask was upscaled to the same dimensions as the cell segmentation masks and used to extract the nearest surface normal for each cell. To obtain the surface normal, a surface mesh was extracted using scikit-images marching cubes, with a step size of 40 and level 0. For all surface meshes, the pymeshfix clean_from_arrays function was used to clean the surface mesh. For the organoid surface mesh, the filter_taubin function from trimesh with 50 iterations was used to smooth the surface. spatial.cKDTree from scipy (v.1.8.1) was used to obtain the nearest surface normal from the centroid of each cell. The absolute cosine similarity from scikit-image between the nearest surface normal and the major axis of each ellipsoid was calculated. We defined the alignment index as the absolute cosine similarity between the major axis of the ellipsoid and the nearest surface normal of the organoid. For Fig. 3n,o, only actin was considered, whereas for Extended Data Fig. 7g, all demultiplexed markers were used. All plots for Fig. 3n and Extended Data Fig. 7g were created using the ellipsoid_plot (https://github.com/aleksandrbazhin/ellipsoid_fit_python).

#### Tracking

All tracking was done using motile (https://github.com/funkelab/motile). First, detections were created for each to be tracked mask labels to create unique identifiers for each label. After this, a candidate graph was built by adding nodes to a networkX graph and noting the centroid location of each mask, accounting for anisotropy as well as adding a node feature for a minimum volume cut-off. The node feature was calculated: 1 – (min_vol_/volume). After the candidate graph was built, edges between the cells of two successive cells were added to the graph. Only edges, where the distance between the centroids of the objects were closer than a maximum distance cut-off, were added. Any edges with a zero weight were removed. After this, the edges were added to the networkX graph and the graph was converted to a motile candidate graph. Motile was then used to initiate a solver. The node and the edge selection weight were then set to −1 and added as costs. Furthermore, appear and disappear costs, as well as maximum number of parents and children constraints were added. Using this, an optimal tracking solution was then solved for and the candidate graph converted to the track graph by removing any edge or node with a solution value of less than 0.5.

#### Lumen tracking

The approach as described above was used to track all 16 movies used in Fig. 2. The lumen were tracked at one-quarter resolution, with a maximum distance of 1,110 μm. The edge metric used was the maximum intersect between two objects *i* and *j*: max(overlap_*ij*_/volume_*i*_,overlap_*ij*_/volume_*j*_). Furthermore, appear and disappear costs of 0.8, as well the constraints for a maximum of 2 children and 2 parents were added. The fusion events were extracted from the solution adjacency graph and used to calculate the number of fusion events per time point. For each movie, the number of lumen fusion over time was then smoothed using a rolling mean of 10, with a minimum period of 1. To visualize the lumen tracks, the organoids were rendered as described above. To colour the 3D renderings to show the number of hours since the fusion event, the nodes of the organoids that have just fused were extracted and then the shortest path length from every lumen to the next fusion event, up to 10 time points away, in the nx graph was calculated.

#### Cell tracking

For the cell tracking, every time point of the green channel, containing actin cells in three movies, was segmented before registration. Then, the green channel was registered as described above, and the translations were applied to the cell masks and the organoid image. A cell track graph was built for each of these movies. The maximum distance used was 24.3 μm, any mask below 1,444 μm^3^ was discarded and the minimum volume node feature was set to 481.6 μm^3^. The edge metric used was the average between the distance between the centroids and the IOU of the label within their bounding boxes. The distance metric was defined as 1 – (dist/max_dist_): the distance between the centroid of two cells within the maximal distance. For the IOU of labels within the bounding box, the mask of cells within the neighbourhood were extracted, padded to equal size and then the IOU between the cell’s masks were calculated. To create the tracklets, the maximum number of children and parents was set to one.

For calculating the net coordinated movement of the cells, the centre of mass from scipy of the segmentations, as well as the velocity vector of the tracks at this time point up to a length of 24 h were calculated. Then the cosine of the angle was calculated between the vector from the centre of mass to the centroid of the cell and the velocity vectors, where movement along the vector away from the centroid was 1 and movement towards the centre of gravity was −1.

The stream plot was created by analysing the directionality of the tracks in the current frame up to 24 h ago. Where a 2D representation of the movement of the tracks up to 24 h ago was calculated for each track at the centroid of each tracked cell. Then, a Gaussian smoothing filter of size 20 was used to smooth out the *u*, *v* and *w* velocity arrays. The angle in *z* was calculated as the arctan2 of *w* and the square root of the *u* and *v* velocity vectors, and the angle in *xy* was calculated using the arctan2 between *u* and *v*. Both of these angle arrays were then normalized to between 0 and 1. A colour array was constructed by using a 2D custom colour map that included the colours of the different directions, as well as each colour on a colour gradient from white to the *xy* colour to black for the *z* direction. Finally, all of this was combined into a stream plot.

The quiver plot was created similarly. For each track at a given time point, the velocities *u*, *v* and *w* from the track in the current frame up to 24 h ago in *x*, *y* and *z* were calculated. After this, the angles in *z* and *xy* were calculated, and the quiver plot was coloured by the directionality for the four directions in *xy* and again from white to the *xy* colour to black in *z*. For the Supplementary Video 9, the quiver plot was overlaid onto the GFP channel for every time point. To calculate the velocity components, the centroids of the tracks were smoothed with a rolling mean of 10 and a minimum period of 1. The displayed vectors were calculated using the displacement of the cells up the previous five time points.

### 4i Image analysis

#### 4i Image processing

The raw images were pre-processed on the fly in NISelements. Background subtraction and shading correction were performed for the individual images of the *z*-stack, followed by generating maximum intensity projections. Tiled images were acquired with an overlap of 10% and stitched with the SVI Huygens software (v23.10.0p6; http://svi.nl). Image processing and feature extraction were carried out as previously described^52^ and re-implemented as a stand-alone Python package (phenoscapes). In brief, organoids were segmented within an initial masking step, followed by image registration based on the DAPI signals of all imaged cycles using ITK-Elastix (https://github.com/InsightSoftwareConsortium/ITKElastix), mask refining, cropping and denoising. Furthermore, we added processing modules for speckle removal and background estimation using SMO^77^, with SMO being applied before estimating a background model for respective imaging and elution cycles. Nucleus and cell segmentations were performed using Cellpose^78^, and fluorescence intensity and morphology features were extracted. The added modules work as described in the following section.

Speckles were determined to be bright spots that occurred across multiple channels and cycles. Therefore, for each stain, a Gaussian filter of size 5 and a threshold based on the top 2 percentile intensity were applied. Then, a speckle mask was created for pixels that occur in this filtered top two percentile across all channels or across four successive cycles in the same channel. The speckle masks were used to zero-out any high-intensity regions after background subtraction.

The lumen masks were extracted using the cell segmentation of the CTNNB1 stain. First, the labelled cell segmentations were binarized and combined with the speckle masks using binary fill holes. The masks were then smoothed using a Gaussian filter with size 20 and then binarized again. Any small holes (less than 238 µm^2^) within the organoid were then removed. Any background pixels within the organoid were considered to be lumen. The contours of both the organoid and the lumen masks were extracted. The distance from each cell to the nearest surface or lumen contour was calculated using KDTree from Scipy. Regionprops was used to extract the major axis length of each lumen.

The codebase for the re-implemented image processing pipeline is available (https://github.com/quadbio/morphodynamics_human_brain_organoid/phenoscapes).

#### 4i Processing for the agarose and compartment analysis

The pipeline, as described above, was run with the following modifications. Before running the pipeline, individual organoids were extracted from the region of interest using a modified version of the simple masking module of phenoscapes, where connected components were used to extract all organoid masks and the bounding boxes surrounding these masks. The bounding box was expanded up to 400 pixels. Small organoids (less than 26,406.25 μm^2^) were excluded from further processing. Before calculating the alignment, the brightest 2% of the image was masked out. For the refined masking step, only the DAPI signal and a Gaussian filter with a sigma of 50 were used. For illustrating in Fig. 4, the staining image of ARL13B was bi-linearly downscaled to 1/10 resolution.

#### Yap stain qualifications

The pipeline was run as described above, with the following modifications. As described in 4i processing for the compartment analysis, before running the pipeline, individual organoids were extracted and small organoids (less than 26,406.25 μm^2^) were excluded from further processing. The bounding boxes for organoid extraction were expanded up to 128 pixels. For the refined masking step, only the DAPI signal and a Gaussian filter with a sigma of 50, as well as n_binary_operations of 30 were used. Furthermore, a speckle removal using the top three percentile was used. To segment the DAPI channel, the Cyto2 cellpose model with a diameter of 40.8, a flow threshold of 0.8 and a cell probability threshold of −1 was used. The mean and the median nuclear yap stain fluorescence were then quantified.

#### 4i Compartment analysis

We fine-tuned cellpose models to either segment the CTNNB1 stain or the DAPI stain. The fine-tuned cellpose models used a cellprob_threshold set of 0.0 and a flow_threshold of 0.9. We used both the cellular and the nuclear segmentations to extract the three compartments: nuclei, cytoplasm or the ECM niche. For the nuclei and the cytoplasmic compartments, we calculated the IOU between each cell and nuclei within the cellular bounding box and assigned the nucleus with the highest IOU, but at least an IOU of 0.2 to each cell. Any cells without a nucleus or nuclei without a cytoplasm were discarded. For the extracellular compartment, the cell segmentations were expanded by 30 pixels and the rest of the cell zeroed out. For each compartment, we extracted features based on the mean intensity. Finally, we only included the measurement of the compartment where the proteins are known to occur. First, we analysed the cytoplasmic and nuclear components. For each stain, we clipped the lower 5th and upper 98th percentiles before further analysis. Any cell with either a nuclear or cellular compartment that is smaller than 50 or a combined size of smaller than 100 pixels was removed. Using Scanpy, we then scaled, calculated the PCA, extracted nearest neighbours with n_pcs set to 10, clustered using the Leiden algorithm with a resolution of 0.9 and extracted the UMAP with a min_dist of 0.2. We annotated the clusters based on the regions and cell types revealed from the dot and feature plots. We overlaid these regions onto the segmented compartments. To analyse the lumen morphology composition, each lumen was designated a region using the most often annotated region of the cells that have been assigned to the lumen and are at most 50 μm away.

The same workflow was applied to the extracellular compartment. However, any extracellular compartment that was smaller than 100 pixels was removed. Using the mean intensity measurement and Scanpy, the PCA was calculated; using 6 principal components, the neighbours were extracted, Leiden clustering was used with a resolution of 0.3 and the UMAP extracted using a min_dist of 0.2. For the analysis, the annotations of the nuclear or cellular compartment were used to add a region identifier to each corresponding extracellular compartment, as well as the abundance of each cluster in each brain region. All violin plots were calculated before the percentile clipping for both conditions and for the proteins in their respective compartment, except for the cytoplasmic ECM components, where the ECM protein intensities were measured in the cytoplasmic compartment.

### Reporting summary

Further information on research design is available in the Nature Portfolio Reporting Summary linked to this article.

## Online content

Any methods, additional references, Nature Portfolio reporting summaries, source data, extended data, supplementary information, acknowledgements, peer review information; details of author contributions and competing interests; and statements of data and code availability are available at 10.1038/s41586-025-09151-3.

## Supplementary information

Supplementary Information Supplementary Methods Table 1, outlining details of the scRNAseq and lightsheet experiments done in this study.

Reporting Summary

Supplementary Information Supplementary Methods Table 2, outlining details of number of organoid sections from different conditions, timepoints and antibodies used for indirect iterative immunohistochemistry (4i).

Supplementary Tables This zipped folder contains Supplementary Tables 1–13, plus legends.

Supplementary Video 1 Sparse and multi-mosaic fluorescently labeled brain organoid imaged for one week. Video shows maximum intensity projections of a developing sparse and multi-mosaic organoid brain from a time lapse dataset of 188-hour lightsheet imaging experiment. Organoids contain 5 different cell lines that contain stable genetic tagging of proteins with red or green fluorescent protein (RFP, GFP), as well as unlabeled cells, showing nuclear membrane (lamin, RFP, magenta), plasma membrane label (CAAX, RFP, magenta), actin (GFP, green), tubulin (RFP, magenta), and nuclei (histone, GFP, green). Timepoint 00:00:00 refers to the onset of imaging. Time is in hours and the scale bar is 100 micrometers.

Supplementary Video 2 3D rotating visualization of a sparse and multi-mosaic organoid. 3D *z*-stack rotations to visualize the dataset in Supplementary Video 1 at 93.5 h (day 7) into imaging.

Supplementary Video 3 Organoid and lumen tissue dynamics in multiple brain organoids imaged in one experiment. Video shows cross sections of 16 different organoids that were imaged together in one experiment. The segmented lumens are false colored with red hot LUT and the organoid tissue is shown in grayscale. Time is in hours and the scale bar is 500 micrometers.

Supplementary Video 4 3D lumen morphodynamics in multiple brain organoids imaged in one experiment. Video shows 3D renderings of segmented lumen in 16 different organoids that were imaged together in one experiment. The experiment includes the organoids shown in supplementary video 1-4. The organoid outlines are shown as light-colored contrast and the lumens are shown in cyan. Time is in hours and the scale bar is 500 micrometers.

Supplementary Video 5 Neuroectoderm development and lumen expansion dynamics in early organoid morphogenesis. Time lapse video showing cross-section of a developing lumen to show changes in lumen and neuroepithelium morphologies over time. The false coloring corresponds to nuclear membrane (lamin, RFP, orange), plasma membrane label (CAAX, RFP, orange), actin (GFP, cyan), tubulin (RFP, orange), and nuclei (histone, GFP, cyan). Time is in hours and the scale bar is 75 micrometers.

Supplementary Video 6 Developing organoids cultured in Matrigel, without any extrinsic matrix and embedded in agarose. Time lapse video showing cross-sections of example neural organoids that were imaged in one experiment and were either embedded in Matrigel, given no-matrix or embedded in agarose. The false coloring corresponds to nuclear membrane (lamin, RFP, orange), actin (GFP, cyan), tubulin (RFP, orange), Time is in hours and the scale bar is 100 micrometers.

Supplementary Video 7 Multi-sample parallel imaging of organoids grown in different extracellular matrix environments. Time lapse video showing cross sections of 16 different organoids that were imaged together in one experiment and were either embedded in Matrigel (first row), given no-matrix (second and third row) or embedded in agarose (fourth row). The segmented lumen are false colored with the red LUT. Organoids were sparse and mosaic, labeled with fluorescent cells showing (lamin, RFP, gray), actin (GFP, gray), tubulin (RFP, gray). Time is in hours and the scale bar is 500 micrometers.

Supplementary Video 8 Tracking 3D lumen fusions across multiple brain organoids cultured in Matrigel, without any extrinsic matrix and embedded in agarose. Video shows 3D renderings of segmented lumen in 16 different organoids showing a timecourse of lumen development in Matrigel, no-matrix and agarose conditions. The experiment includes the organoids shown in supplementary video 6 and 7. The organoid masks are shown as light grey, and the lumen are shown with grey to yellow color scale indicating the hours from the last fusion event with yellow showing the just fused lumen. Time is in hours and the scale bar is 500 micrometers.

Supplementary Video 9 Cell tracks in organoids cultured in Matrigel, without any extrinsic matrix and embedded in agarose. Video shows smoothed cell tracks of actin labelled cells overlaid on maximum intensity projections of a developmental timecourse of organoids grown in Matrigel, no-matrix and agarose. The arrow colors correspond to: white is towards the imaging objective, black away from it, yellow is down, magenta is up, green points left and cyan towards right of the image. Time is in hours and the scale bar is 100 micrometers.

Supplementary Video 10 Organoid developmental dynamics under control and YAP1 activator condition. Video shows maximum intensity projections of two different organoids from timepoint 00:72:00 hour (Day 8) onwards. Control is on the left and an organoid that was treated with YAP1 activator Py-60 on day 7 is on the right. The false coloring corresponds to nuclear membrane (lamin, RFP, orange), actin (GFP, cyan) and tubulin (RFP, orange), Time is in hours and the scale bar is 100 micrometers.

## Data Availability

Raw sequencing data are available at ArrayExpress (accession number E-MTAB-15057). Processed scRNA-seq data are available via Zenodo^79^ (10.5281/zenodo.15236859). AnnData files of the indirect iterative immunohistochemistry (4i) experiment are available via Zenodo^80^ (10.5281/zenodo.15238487). Owing to its large size, the light-sheet data will be made available on request. All experimental materials are available on request from the corresponding authors.

## Code availability

All code generated in the study, including analysis parameters, is available at GitHub (https://github.com/quadbio/morphodynamics_human_brain_organoid) and via Zenodo^81^ (10.5281/zenodo.15149153). All other code is available on request from the corresponding authors.
