# Supplementary material for: Morphodynamics of human early brain organoid development
Source: Nature. 2025 Jun 18;644(8078):1010–9. doi: 10.1038/s41586-025-09151-3 (PMC12390842; doi:10.1038/s41586-025-09151-3)
Supplement: Supplementary file 1 — Supplementary Methods Table 1, outlining details of the scRNAseq and lightsheet experiments done in this study. [file 41586_2025_9151_MOESM1_ESM.pdf]

nature portfolio

https://doi.org/10.1038/s41586-025-09151-3

Supplementary information

Morphodynamics of human early brain organoid development

In the format provided by the
authors and unedited

Supplementary methods table

Serial No.	Experiment dataset	Data type	Timepoint	Figure	Cell lines used	Time resolution
1	Organoid development timecourse (Protocol I)	scRNAseq	Days (5,7,11,16,21,30)	Figure 1,2	Histone2B-mEGFP	-
2	Matrix perturbation (Protocol I)	scRNAseq	Day 13	Extended Data Figure 10	WTC-11	-
3	YAP activator (Protocol I)	scRNAseq	Day 10	Figure 5	WTC-11	-
4	Matrix perturbation (Protocol II)	scRNAseq	Day 16	Extended Data Figure Figure 11	WTC-11	-
5	YAP activator (Protocol II)	scRNAseq	Day 16	Extended Data Figure 12	WTC-11	-
6	Sparse and multi-mosaic organoids	Lightsheet	Days 4-12	Figure 1,2,3	WTC-11 90% + 2% each of: Histone2B-mEGFP , mEGFP-Beta-Actin, mTagRFP–T–CAAX, mTagRFP–T-TUBA1B, mTagRFP–T-LMNB1	30 minutes
7	Matrix perturbation (Protocol I)	Lightsheet	Days 4-12	Figure 2,3	WTC-11 94% + 2% each of: mEGFP-Beta-Actin, mTagRFP–T-TUBA1B, mTagRFP–T-LMNB1	60 minutes
8	YAP activator (Protocol I)	Lightsheet	Days 4-10	Figure 5	WTC-11 94% + 2% each of: mEGFP-Beta-Actin, mTagRFP–T-TUBA1B, mTagRFP–T-LMNB1	60 minutes
9	Mosaic organoid (Protocol I)	Lightsheet	Days 4-19	Extended Data Figure 2	WTC-11 (98%) + 1%: Histone2B-mEGFP, mTagRFP–T–CAAX,	30 minutes
10	NKX2-1:GFP	Lightsheet	Days 0-21	Extended Data Figure 2	HES3 (NKX2-1:GFP)	60 minutes
