# Supplementary material for: Morphodynamics of human early brain organoid development
Source: Nature. 2025 Jun 18;644(8078):1010–9. doi: 10.1038/s41586-025-09151-3 (PMC12390842; doi:10.1038/s41586-025-09151-3)
Supplement: Supplementary file 2 — Reporting Summary [file 41586_2025_9151_MOESM2_ESM.pdf]

nature portfolio

Corresponding author(s): Barbara Treutlein, Akanksha Jain

Last updated by author(s): Apr 18, 2025

# Reporting Summary

Nature Portfolio wishes to improve the reproducibility of the work that we publish. This form provides structure for consistency and transparency in reporting. For further information on Nature Portfolio policies, see our Editorial Policies and the Editorial Policy Checklist.

## Statistics

For all statistical analyses, confirm that the following items are present in the figure legend, table legend, main text, or Methods section.

| n/a | Confirmed | |
|---|---|---|
| ☐ | ☒ | The exact sample size (*n*) for each experimental group/condition, given as a discrete number and unit of measurement |
| ☐ | ☒ | A statement on whether measurements were taken from distinct samples or whether the same sample was measured repeatedly |
| ☐ | ☒ | The statistical test(s) used AND whether they are one- or two-sided<br>*Only common tests should be described solely by name; describe more complex techniques in the Methods section.* |
| ☒ | ☐ | A description of all covariates tested |
| ☐ | ☒ | A description of any assumptions or corrections, such as tests of normality and adjustment for multiple comparisons |
| ☐ | ☒ | A full description of the statistical parameters including central tendency (e.g. means) or other basic estimates (e.g. regression coefficient) AND variation (e.g. standard deviation) or associated estimates of uncertainty (e.g. confidence intervals) |
| ☐ | ☒ | For null hypothesis testing, the test statistic (e.g. *F*, *t*, *r*) with confidence intervals, effect sizes, degrees of freedom and *P* value noted<br>*Give P values as exact values whenever suitable.* |
| ☒ | ☐ | For Bayesian analysis, information on the choice of priors and Markov chain Monte Carlo settings |
| ☒ | ☐ | For hierarchical and complex designs, identification of the appropriate level for tests and full reporting of outcomes |
| ☒ | ☐ | Estimates of effect sizes (e.g. Cohen's *d*, Pearson's *r*), indicating how they were calculated |

*Our web collection on statistics for biologists contains articles on many of the points above.*

## Software and code

Policy information about availability of computer code

| | |
|---|---|
| Data collection | A detailed description of data collection is provided in the methods. |
| Data analysis | A detailed description of data analysis is provided in the methods.. All code generated in the study including analysis parameters is available at GitHub (https://github.com/quadbio/morphodynamics_human_brain_organoid) and via Zenodo (10.5281/zenodo.15149153). All other codes are available upon request. Data analysis software/tools/algorithms/packages are as follows:<br>* SVI Huygens (v23.10.0p6)<br>* Cell Ranger (v.3.0.2)<br>* Seurat R package (v.4.3.0)<br>* STAR (v2.7.11b)<br>* scanpy (v1.10.0)<br>* Motile (v0.2.0)<br>* Morphometrics (v0.0.6)<br>* Embedseg (v0.2.5)<br>* Noise2Void (v0.3.1)<br>* Scipy (v1.7.3)<br>* LStree (v0.1)<br>* scikit-learn (v0.18.3)<br>* scikit-image (v1.1.1)<br>*R version 4.4.0 |

For manuscripts utilizing custom algorithms or software that are central to the research but not yet described in published literature, software must be made available to editors and reviewers. We strongly encourage code deposition in a community repository (e.g. GitHub). See the Nature Portfolio guidelines for submitting code & software for further information.

# Data

Policy information about availability of data

All manuscripts must include a data availability statement. This statement should provide the following information, where applicable:

- Accession codes, unique identifiers, or web links for publicly available datasets
- A description of any restrictions on data availability
- For clinical datasets or third party data, please ensure that the statement adheres to our policy

Raw sequencing data is available at ArrayExpress (accession number: E-MTAB-15057). Processed scRNAseq data is available via Zenodo (10.5281/zenodo.15236859). AnnData files of the Indirect iterative immunohistochemistry (4i) are available via Zenodo (10.5281/zenodo.15238488). Due to its large size the light sheet data will be made available upon request. All experimental materials are available upon request to akanksha.jain@bsse.ethz.ch and barbara.treutlein@bsse.ethz.ch.

# Research involving human participants, their data, or biological material

Policy information about studies with human participants or human data. See also policy information about sex, gender (identity/presentation), and sexual orientation and race, ethnicity and racism.

| | |
|---|---|
| Reporting on sex and gender | We have used a female human ES cell line (HES-3), which has been established previously in 2018 by ES Cell International Pte Ltd. in Singapore. Information about this hES cell line can be found under https://hpscreg.eu/cell-line/ESIBIe003-A. |
| Reporting on race, ethnicity, or other socially relevant groupings | The information on race or ethnicity is not available for the human ES cell line HES-3 and was not relevant for our study. |
| Population characteristics | see above. |
| Recruitment | No human participants were recruited for this study. The Murdoch Children's Research Institute and Monash University kindly provided us the HES-3 NKX2-1:GFP cell line. The HES-3 human ES line is registered in hPSCreg.eu (https://hpscreg.eu/cell-line/ESIBIe003-A). |
| Ethics oversight | We have not recruited any human participants for this study. The human ES cell line HES-3 was previously generated with consent by the donor as specified here: https://hpscreg.eu/cell-line/ESIBIe003-A |

Note that full information on the approval of the study protocol must also be provided in the manuscript.

# Field-specific reporting

Please select the one below that is the best fit for your research. If you are not sure, read the appropriate sections before making your selection.

☒ Life sciences ☐ Behavioural & social sciences ☐ Ecological, evolutionary & environmental sciences

For a reference copy of the document with all sections, see nature.com/documents/nr-reporting-summary-flat.pdf

# Life sciences study design

All studies must disclose on these points even when the disclosure is negative.

| | |
|---|---|
| Sample size | Initially 16 organoids were profiled with live imaging, and a high degree of consistency in morphodynamic behaviour was identified. Following this, 4 replicates were chosen for control or treatment imaging experiments. No separate sample sample size calculations were performed. For scRNAseq experiments, we dissociated several organoids (n>3) depending on timepoint and treatment to obtain cell suspensions (see methods) which gave 10k-30k cells recovery with the 10xv3.1. |
| Data exclusions | No datasets were excluded from the study. For single cell analysis we performed strict quality filtering and excluded individual cells that did contain high quality data. For the details and filtering criteria see methods. Quality control on tissue segmentation: small lumen segmentations were removed. Quality control on single cell segmentation: removal of dim/small segmentations and removal of wrongly segmented cells though a manually trained classifier. |
| Replication | We used fluorescent cell lines based on the parental WTC-11 iPSC line and multiple organoids for each timepoint of the timecourse and the perturbation experiments. For lightsheet live imaging, 16 organoids were imaged in parallel as 16 biological replicates with 4 media divisions. Control/perturbation experiments were done with at least 4 organoid replicates per condition. In each scRNAseq experiments several thousands of cells were recovered in each condition, which serve as biological replicates per cell type/cluster. The number of cells recovered after filtering and quality control in each experiment are listed in methods section "Preprocessing of scRNA-seq data from the organoid timecourse" and "Preprocessing of scRNA-seq data from all other datasets." All attempts at replication were successful. |
| Randomization | Experiments were not randomized. We quantified effects in at least two or more organoid batches with complementary quantitative measurement techniques (scRNAseq or qPCR or fixed stainings to validate the results across multiple organoid batches). For each experiment |

we compared measurements of several organoids between control and pertubation conditions and compared measurements in n>100 cells in control vs perturbation conditions.

Blinding

Data collection:
Organoids were generated from embryoid bodies and given different treatments between control and perturbations. Organoids for each scRNAseq experiment were picked blinded without any prescreening. Several organoids from each treatment were pooled together to create single-cell suspensions. For live imaging, embryoid bodies were pre-screened with an epifluorescence microscope to ensure both GFP and RFP fluorescent signal was present before using them for light sheet imaging and not selected blinded. Organoids to be imaged as control or treatment conditions were selected blinded in the imaging chamber followed by allocation as control or treatment condition whithin separate subchambers. The imaging was performed simultaneously for all organoids in an unbiased manner.

Data analysis:
Data analysis was performed blinded. We performed an unbiased analysis without prior hypothesis and quantified across all samples together. Only when visualizing quantifications of the data, the individual control and perturbation conditions and their respective effects were revealed.

# Reporting for specific materials, systems and methods

We require information from authors about some types of materials, experimental systems and methods used in many studies. Here, indicate whether each material, system or method listed is relevant to your study. If you are not sure if a list item applies to your research, read the appropriate section before selecting a response.

## Materials & experimental systems

| n/a | Involved in the study |
|---|---|
| ☐ | ☒ Antibodies |
| ☐ | ☒ Eukaryotic cell lines |
| ☒ | ☐ Palaeontology and archaeology |
| ☒ | ☐ Animals and other organisms |
| ☒ | ☐ Clinical data |
| ☒ | ☐ Dual use research of concern |
| ☒ | ☐ Plants |

## Methods

| n/a | Involved in the study |
|---|---|
| ☒ | ☐ ChIP-seq |
| ☒ | ☐ Flow cytometry |
| ☒ | ☐ MRI-based neuroimaging |

## Antibodies

Antibodies used

See Methods, in the sections "Immunohistochemistry", "Bulk Cut&Tag for YAP1" and "Indirect iterative immunohistochemistry (4i)".
List of antibodies:
Antibody Host Dilution Supplier Catalog Nr.
Anti-GFP FITC Goat 1/250 Abcam ab6662
Arl13b Rabbit 1/500 ProteinTech 17711-1-AP
B-cat Mouse 1/200 BD-Bio 610154
CDH1 (e-Cad) Goat 1/500 Novus AF648
Col2A1 Mouse 1/1000 OriGene Tech BM332
Col4A1 Goat 1/25Merck AB769
Col4A1 Mouse 1/500 Sigma C1926
CTIP2 Rat 1/500 Abcam ab18465
Cytokeratin 18 Mouse 1/200 Abcam ab668
DLL1 Goat 1/250 Abcam ab85346
DLX2 Mouse 1/200 SantaCruz sc-393879
Fibronectin Rabbit 1/250 Abcam ab2413
FilaminA Rabbit 1/500 Invitrogen PA5-82043
FoxG1 Rabbit 1/500 Abcam ab18259
Gbx2 Goat 1/250 LSBio LS-B3947-50
Gli3 Goat 1/300 R&D AF3690
GPC3 Sheep 1/100 Abnova PAB9896
GPR177/WLS Rabbit 1/300 ProteinTech 17950-1-AP
GSX2 Rabbit 1/200 Millipore abn162
HAPLN1 Goat 1/500 Novus AF2608
HES1 Rabbit J1/6400 Cell Signalling 11988
HES4 Sheep 1/200 R&D AF3600
HOXB1 Sheep 1/200 R&D AF6318
Human Integrin Alpha V beta 5 Mouse 1/200 R&D MAB2528
IGFBP2 Rabbit 1/200 NSJ Bioreagents NSJ-F51220
IRX3 Mouse 1/300 LSBio LS-B6712
ITGA5B1 Rat 1/100 Sigma MAB2575
ITGA6 Rat 1/200 Sigma MAB1378
ITGB5 Sheep 1/100 R&D AF3824-SP
JAG1 Goat 1/100 Novus AF1277
Laminin Rabbit 1/300 Abcam ab11575
n-Cad Sheep 1/100 R&D AF6426

NES Rabbit 1/100 Merck N5413
NKX2-1 Mouse 1/200 Invitrogen MA5-13961
NR2F1/COUP Rabbit 1/200 Abcam ab181137
NRG1 Mouse 1/200 Thermo Fisher MA5-12896
NUMB Goat 1/200 Abcam ab4147
Oct.04 Mouse 1/500 Abcam ab184665
OTX2 Mouse 1/500 Thermo Fisher MA5-15854
PAX3 Goat 1/200 Thermo Fisher PA5-19271
PAX3/7 Mouse 1/250 SantaCruz sc-365843
PAX6 Sheep 1/100 R&D AF8150-SP
Phospho histone S3,Mouse,1/500,Cell signalling,#9706
Piezo1 Rabbit 1/200 ProteinTech 15939-1-AP
PROM1/CD133 Rabbit 1/1000 Abcam ab216323
p-VIM (Ser55) Mouse 1/500 MBL Int D076-3
p-YAP1 Rabbit 1/200 Cell Signalling Tech 4911S
RAX Guinea Pig 1/200 Takara Bio M229
RSPO3 Rabbit 1/100 Abcam ab233113
Scribble Rabbit 1/200 Cell Signalling Tech 44755
SFRP2 Goat 1/200 LSBio LS-C61791-100
SIX3 Mouse 1/200 Abnova H00006496-M04
SOX10 Rabbit 1/100 Abcam ab227680
Sox2 Rabbit 1/100 Sigma AB5603
Sox21 Goat 1/200 R&D AF3538
TBR1 Rabbit 1/500 Abcam ab31940
TBR2 Chicken 1/200 Millipore AB15894
Tubulin-B3 Mouse 1/1000 BioLegend MMS-435P
Vangl2 Sheep 1/200 R&D AF4815
VCAN Goat 1/100 Novus AF3054
VIM Chicken 1/500 Sigma AB5733
Vinculin Rabbit 1/100 R&D MAB2528
WNT5A Rabbit 1/1000 Abcam ab235966
WWTR1 Rabbit 1/300 Sigma HPA007415
Yap1 Mouse 1/400 Abnova H00010413-M01
YAP1 Rabbit 1/200 Abcam ab52771

Secondary Antibody list
Antibody Host Dilution Supplier Catalog Nr.
Donkey-a-Rabbit AlexaFluor Plus 488 Donkey 1/500 Thermo Scientific A32790
Goat-a-rat AlexaFluor 488 Goat 1/500 Thermo Scientific A21212
Goat-a-Rabbit AlexaFluor 488 Goat 1/500 Thermo Scientific A11008
Donkey-a-Rat AlexaFluor Plus 488 Donkey 1/500 Thermo Scientific A48269
Goat-a-Rabbit AlexaFluor 640 Goat 1/500 Thermo Scientific A21245
Donkey-a-mouse AlexaFluor Plus 555 Donkey 1/500 Thermo Scientific A32773
Donkey-a-rabbit AlexaFluor 568 Donkey 1/500 Thermo Scientific A10042
Goat-a-mouse AlexaFluor 568 Goat 1/500 Thermo Scientific A21124
Goat-a-Chicken AlexaFluor 640 Goat 1/500 Thermo Scientific A21449
Donkey-a-Sheep AlexaFluor 640 Donkey 1/500 Thermo Scientific A21448
Donkey-a-goat AlexaFluor 640 Donkey 1/500 Thermo Scientific A21447
Goat-a-GuineaPig AlexaFluor 640 Goat 1/500 Thermo Scientific A21450
Donkey-a-Rabbit 568 Donkey 1/500 Abcam ab175470

Validation

See Methods, all antibodies used in this study are commercially available and have been validated by the manufacturer. Antibody dilution and signal localization validation was estimated with staining experiments performed before the 4i experiment that is reported in this study. We performed additional QC during image analysis to determine the expected antibody signal and sub-cellular or extracellular localization behavior expected based on manufacturere's specification and reported localizations of the proteins (Genecards).

# Eukaryotic cell lines

Policy information about cell lines and Sex and Gender in Research

Cell line source(s)

See Methods, in the section "Stem cell and organoid culture". We used the following iPSC cell line for all experiments (also see Supplementary methods table 1) : Histone2B-mEGFP that uniformly labels nuclei (cell line ID: AICS 0061 036, cl.036), mEGFP-Beta-Actin that uniformly labels ACTB (cell Line ID: AICS-0016-184 cl.184), mTagRFP–T–CAAX which labels cell membrane (cell line ID: AICS-0054-091, cl.091), mTagRFP–T-Tubulin-alpha1b that labels TUBA1B (cell line ID: AICS 0031 035, cl.035), mTagRFP–T-LaminB1 that labels LMNB1 (cell line ID: AICS 0034 062, cl.062) and unlabeled WTC iPSCs (cell line ID GM25256). NKX2.1-GFP/w hESCs were obtained from Agnete Kirkeby's research group at the University of Copenhagen, after the arrangement of an MTA with Prof. Ed Stanley and Prof. Andrew G. Elefanty (Murdoch Childrens Research Institute, Melbourne)

Authentication

We used commercial cell lines provided by the Coriell Institute (see Methods).The Coriell institute performs a Karyotype analysis as a routine part of the quality control process to address the genomic stability of the lines. These results are available in the Certificate of Analysis for each line here on the Coriell site and are also in the Cell Catalog on the Allen Cell Explorer website, allencell.org. WTC-TUBA1BmTAgRFP-T (cell line ID: AICS 0031 035, cl.035) was used to create the WLS-knockout line. The control line and WLS-KO line used in this study were karyotyped and showed a normal karyotype.

| | |
|---|---|
| Mycoplasma contamination | Cell lines were tested for mycoplasma contamination on a regular basis using a PCR-based test and were found to be negative for mycoplasma. |
| Commonly misidentified lines<br>(See ICLAC register) | None. |
