# Supplementary material for: Morphodynamics of human early brain organoid development
Source: Nature. 2025 Jun 18;644(8078):1010–9. doi: 10.1038/s41586-025-09151-3 (PMC12390842; doi:10.1038/s41586-025-09151-3)
Supplement: Supplementary file 3 — Supplementary Methods Table 2, outlining details of number of organoid sections from different conditions, timepoints and antibodies used for indirect iterative immunohistochemistry (4i). [file 41586_2025_9151_MOESM3_ESM.pdf]

Overview of organoids used for 4i analysis:

Conditions	No. of sectiones used for analysis	Timepoints
Matrigel	2	Day 7
Matrigel	3	Day 15
Matrigel	4	Day 21
No Matrix	2	Day 7
No Matrix	4	Day 15
No Matrix	3	Day 21
Agarose	2	Day 7
Agarose	3	Day 11

Antibodies tested

Category	List	Worked/ used in analysis
Polarity and junction markers	CDH1, CDH2, ARL13B, ITGAVBV, ITGB5, ITGA6, ITGB1 , ITGA5B1, VINC, VANGL2, SCRIB	CDH1, CDH2, ARL13B, ITGAVBV, ITGB5, ITGB1 , ITGA5B1, VINC, VANGL2, SCRIB
ECM proteins	IGFBP2, HAPLN1, mCOL4A1, hCOL4A1, COL2A1, LAMA1, FN1, GPC3, VCAN	IGFBP2, HAPLN1, mCOL4A1, COL2A1, LAMA1, FN1, GPC3, VCAN
Transcription factors/ Cell type markers	DLX2, RSPO3, GBX2, SOX10, RAX, FOXG1, PAX6, OCT4, IRX3, PAX3/7, SIX3, OTX2, NR2F1/COUP=TFI, SOX21, SOX2, HOXB1, TBR1, CTIP2, NKX2-1, GLI3, PAX3, TBR2, GSX2	RSPO3, GBX2, SOX10, RAX, FOXG1, PAX66, OCT4, PAX3/7, SIX3, OTX2, NR2F1/COUP=TFI, SOX21, SOX2, TBR1, GLI3, PAX3, TBR2, GSX2
Signalling pathway	GPR177/WLS, YAP1, SRFP2, WNT5A, HES1, HES4, JAG1, CTNNB1, DLL1, Phospho-YAP1, NRG1, WWTR1/TEAD1	GPR177/WLS, YAP1, SRFP2, WNT5A, HES1, HES4, JAG1, CTNNB1, DLL1
Miscellaneous: Cytoskeletal/ cell type markers	CK18, VIM, pVIM , NES, PIEZO1, TUBB3, PROM1/CD133, PH3S0, FLNA, NUMB	CK18, VIM, pVIM , NES, PIEZO1, TUBB3, PROM1/CD133, PH3S0, FLNA, NUMB
