# Supplementary material for: Morphodynamics of human early brain organoid development
Source: Nature. 2025 Jun 18;644(8078):1010–9. doi: 10.1038/s41586-025-09151-3 (PMC12390842; doi:10.1038/s41586-025-09151-3)
Supplement: Supplementary file 4 — This zipped folder contains Supplementary Tables 1–13, plus legends. [file 41586_2025_9151_MOESM4_ESM.zip › 2023-07-12919C-Supplementary Tables/Supplementary Table legends.pdf]

Supplementary Table 1: Gene list of pseudotime dependent genes from day 5 to day 11.

Supplementary Table 2: Gene ontology term analysis list of pseudotime dependent genes.

Supplementary Table 3: Differentially expressed genes between matrigel and no matrix organoids from an scRNAseq dataset sequenced on day 13.

Supplementary Table 4: Gene ontology term analysis list for genes upregulated in matrigel (day 13).

Supplementary Table 5: Gene ontology term analysis list for genes upregulated in no matrix (day 13).

Supplementary Table 6: Differentially expressed genes between matrigel and no matrix organoids from an scRNAseq dataset sequenced on day 16.

Supplementary Table 7: Differentially expressed genes between Control and YAP1 activator treated organoids from an scRNAseq dataset sequenced on day 10.

Supplementary Table 8: Differentially expressed genes between Control and YAP1 activator treated organoids from an scRNAseq dataset sequenced on day 16.

Supplementary Table 9: Differentially expressed genes between Control and WLS-KO organoids treat with Chiron and used for scRNAseq

Supplementary Table 10: Differentially expressed genes between Control and WLS-KO organoids treat with Py-60 and used for scRNAseq

Supplementary Table 11: List of antibodies with dilutions and source used for indirect iterative immunohistochemistry (4i).

Supplementary Table 12: Detailed composition of buffers used for indirect iterative immunohistochemistry (4i).

Supplementary Table 13: List of p-values resulting from statistical significance testing of comparative analyses presented in Figures 4h, 4j, 5b, 5e, Extended figure 3d, Extended figure 3f, Extended Figure 10b, Extended Figure 10e.
